# Endotoxemia-mediated activation of acetyltransferase P300 impairs insulin signaling in obesity

**DOI:** 10.1038/s41467-017-00163-w

**Published:** 2017-07-25

**Authors:** Jia Cao, Jinghua Peng, Hongying An, Qiyi He, Tatiana Boronina, Shaodong Guo, Morris F. White, Philip A. Cole, Ling He

**Affiliations:** 1 Division of Metabolism, Department of Pediatrics, Johns Hopkins University School of Medicine, Baltimore, MD 21287 USA; 2 Department of Medicine, Southern Medical University, Guangzhou, 510515 China; 3 Department of Neuroscience, Brown University, Providence, RI 02912 USA; 4 Departments of Biochemistry, Johns Hopkins University School of Medicine, Baltimore, MD 21287 USA; 5 Department of Nutrition and Food Science, Texas A&M University, College Station, TX 77843 USA; 6 Division of Endocrinology, Boston Children’s Hospital, Harvard University, Cambridge, MA 02115 USA; 7 Department of Pharmacology and Molecular Sciences, Johns Hopkins University School of Medicine, Baltimore, MD 21287 USA

## Abstract

Diabetes and obesity are characterized by insulin resistance and chronic low-grade inflammation. An elevated plasma concentration of lipopolysaccharide (LPS) caused by increased intestinal permeability during diet-induced obesity promotes insulin resistance in mice. Here, we show that LPS induces endoplasmic reticulum (ER) stress and protein levels of P300, an acetyltransferase involved in glucose production. In high-fat diet fed and genetically obese *ob/ob* mice, P300 translocates from the nucleus into the cytoplasm of hepatocytes. We also demonstrate that LPS activates the transcription factor XBP1 via the ER stress sensor IRE1, resulting in the induction of P300 which, in turn, acetylates IRS1/2, inhibits its association with the insulin receptor, and disrupts insulin signaling. Pharmacological inhibition of P300 acetyltransferase activity by a specific inhibitor improves insulin sensitivity and decreases hyperglycemia in obese mice. We suggest that P300 acetyltransferase activity may be a promising therapeutic target for the treatment of obese patients.

## Abstract (web summary)

Elevated plasma LPS levels have been associated with insulin resistance. Here Cao et al. show that LPS induces ER stress and P300 activity via the XBP1/IRE1 pathway. P300 acetylates IRS1/2 and inhibits its binding with the insulin receptor. The consequent impairment of insulin signaling can be rescued by pharmacological inhibition of P300.

## Introduction

Insulin resistance is a hallmark of obese and type 2 diabetic patients^1, 2^. Defective insulin sensitivity in the liver results in increased glucose production, which is the major cause of hyperglycemia in diabetic patients^3–5^. The deacetylase Sirtuin1 has been documented to upregulate insulin signaling through several mechanisms, including deacetylation of the insulin receptor substrate^6–8^. This evidence indicates that acetylation of mediators/regulators in the insulin signaling pathway downregulates insulin signaling. However, the identity of the acetyltransferase that modulates insulin signaling remains unclear.

The endoplasmic reticulum (ER) is at the intersection of inflammation and metabolic diseases^9–11^. ER stress can induce the ‘unfolded protein response’ through the activation of three canonical pathways: IRE1-XBP1s, PERK-eIF2 and ATF6 (refs ^12, 13^). Solid evidence has demonstrated an elevation of ER stress in the liver of obese and diabetic patients^14^, whereas administration of an ER stress inhibitor to *ob/ob* mice normalized blood glucose levels^15^. These studies suggest a critical role of ER stress in the development of insulin resistance^16, 17^. Obese individuals and animals fed a high-fat diet (HFD) experience a change in gut microbiota composition as well as a 2–3-fold increase in blood lipopolysaccharide (LPS) levels^18–22^, and a low-dose LPS infusion results in hepatic insulin resistance in a mouse model^18^. The impairment of hepatic insulin signaling by LPS might be in part through the activation of ER stress. Here we show that HFD feeding rapidly increases LPS levels in the liver, which induces P300 through the IRE1-XBP1 pathway in the ER stress response. Subsequently, P300 disrupts insulin signaling by acetylating insulin receptor substrate (IRS)1/2, resulting in decreased association of IRS with the insulin receptor β-subunit (IRβ). Therefore, the activation of P300 acetyltransferase activity appears to be one of the prime factors in the development of hepatic insulin resistance in obesity.

## Results

### The induction of acetyltransferase P300 in the liver of obese mice

**Fig. 1:**
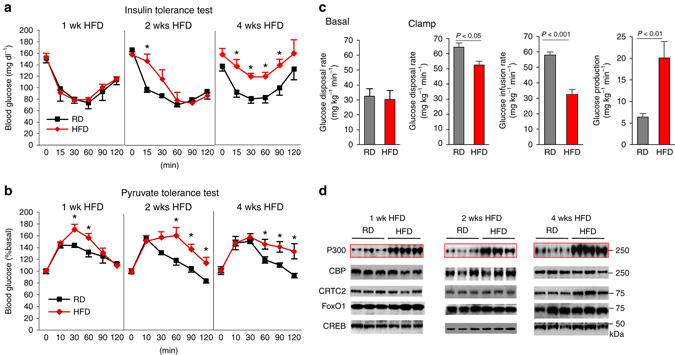
HFD feeding results in P300 induction in the liver. **a**, **b** Insulin tolerance test (4 h fasting, 0.8 unit per kg, *n* *=* 4) **a** and pyruvate tolerance test (5 h fasted, 2 g kg^−1^, *n* *=* 4) **b** in C57BL/6 mice fed a regular diet or HFD for 1–4 weeks. **c** WT mice were fed a regular diet or an HFD for 2 weeks. Basal glucose production in mice after 5 h fasting (*left*) and glucose disposal rates, glucose infusion rates, and hepatic glucose production during the clamp experiment (*n* *=* 4–5). **d** Liver tissues were harvested in mice after feeding on an HFD for 1, 2 and 4 weeks, each lane represents a mouse sample. Data are presented as mean ± s.e.m. Statistical significance was calculated with a Student’s *t*-test

A high-fat, western-style diet is an important predisposing factor for the onset of diabetes and obesity. Mice developed insulin resistance 2 weeks after consuming an HFD (60% calories from fat) along with increased glucose production (Fig. 1a–c and Supplementary Fig. 1a). Considering that acetyltransferase P300 and CBP are critical co-activators in the regulation of hepatic glucose production^23, 24^, we determined the protein levels of these co-activators in the liver of mice fed an HFD. Hepatic P300 protein levels increased dramatically 1 week after feeding the HFD and before the onset of insulin resistance; however, the mRNA levels of P300 did not change significantly (Fig. 1d and Supplementary Fig. 1b), suggesting that the induction of P300 occurred at the post-transcriptional level. P300 protein levels in muscle and adipose tissues did not change significantly after HFD feeding (Supplementary Fig. 1c, d).

### LPS induces acetyltransferase P300 in hepatocytes

**Fig. 2:**
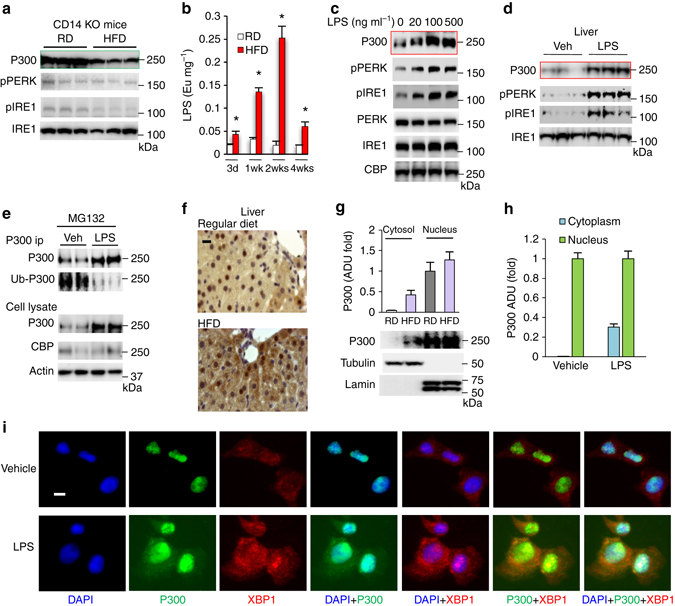
Elevation of hepatic LPS levels by HFD feeding leads to P300 induction. **a** CD14 knockout mice were fed on a regular chow diet or an HFD for 2 weeks. **b** LPS levels in the liver of mice fed an HFD for up to 4 weeks (*n* *=* 4). Each bar represents the mean ± s.e.m. *, *P* **<** 0.05, Student’s *t*-test. **c** Hepa1-6 cells were treated with indicated amount of LPS for 24 h. **d** LPS (0.5 μg per g of body weight per day) was administrated to C57BL/6 mice through intraperitoneal injection for 2 weeks. **e** Hepa1-6 cells were treated with vehicle or LPS (500 ng ml^−1^) for 24 h, followed by the treatment with (20 µM) MG132 for 4 h. Cell lysates were incubated with antibody against P300 (16 h, 4 °C). **f** C57BL/6 mice were fed on an HFD for 2 weeks, frozen liver tissues were sectioned and immunostained with P300 antibody. Scale bar, 20 µm. **g** Cytoplasmic and nuclear extracts were prepared from the liver of mice fed a regular diet or HFD for 2 weeks (*n* *=* 3). **h** Densitometric analysis of P300 in the cytoplasm and nucleus in hepatocytes treated with LPS as in Fig. 2i (*n* = 8). **i** Forty-eight hours after the treatment with LPS (50 ng ml^−1^), Hepa1-6 cells were subjected to immunofluorescence staining. Scale bar, 10 µm. **a**, **d** Each lane represents a mouse sample

We found that HFD feeding could not induce P300 protein levels in the liver of CD14 knockout mice and did not affect glucose production in the liver in CD14 knockout mice (Fig. 2a, Supplementary Fig. 1e). Since HFD feeding leads to 2–3-fold increase in blood LPS levels^18, 19^ and LPS-initiated inflammation occurs through the CD14/TLR4 pathway^25, 26^, we determined LPS levels in the liver of mice fed an HFD for up to 4 weeks. HFD feeding elevated hepatic LPS levels, which peaked after 2 weeks of HFD feeding (Fig. 2b). Next, we tested whether LPS is able to induce P300 in hepatocytes. As shown in Fig. 2c, LPS increased P300 protein levels and induced ER stress. Moreover, intraperitoneal injection of LPS induced P300 protein in the liver (Fig. 2d). We further determined the ubiquitination levels of P300 in Hepa1-6 cells treated with LPS, followed by the treatment with proteasome inhibitor MG132. LPS significantly decreased ubiquitin-conjugated P300 and increased P300 protein levels (Fig. 2e), indicating that LPS mediates P300 induction by decreasing its ubiquitination and degradation. P300 is mainly a nuclear protein; however, substantial amounts of P300 proteins were located in the cytoplasm in the liver of mice fed an HFD and *ob/ob* mice (Fig. 2f, g and Supplementary Fig. 1f). P300 is a nuclear protein in untreated cells, but LPS treatment led to the cytoplasmic localization of P300 in hepatocytes (Fig. 2h, i and Supplementary Fig. 1g).

### Activation of the IRE1-XBP1s pathway leads to P300 induction

**Fig. 3:**
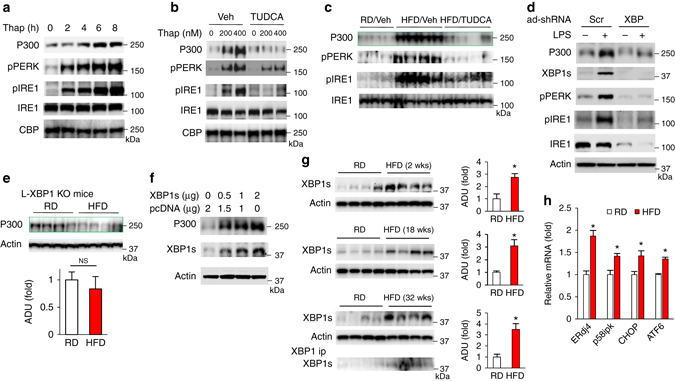
Activation of the IRE1-XBP1s pathway leads to P300 induction and cytoplasmic distribution. **a** Hepa1-6 cells were treated with 300 nM thapsigargin and harvested at indicated time points. **b** Hepa1-6 cells were pretreated with TUDCA (500 μg ml^−1^) for 1 h, then, treated with indicated amounts of thapsigargin for 4 h. **c** Mice were fed an HFD along with the treatment of TUDCA (500 mg kg^−1^ d^−1^ i.p.) for 2 weeks. RD, regular diet. Veh, vehicle. **d** Immunoblots of lysates from Hepa1-6 cells treated with SCR or XBP1 adenoviral shRNAs for 24 h followed by treatment with LPS for 24 h. **e** Homozygous floxed XBP1 mice were injected with AAV8-TBG-Cre (1 × 10^11^ GC/mouse) through the jugular vein, and fed on an HFD for 2 weeks. (*lower*) Densitometric analysis of P300 in the liver. **f** Hepa1-6 cells were transfected with equal amounts of expression plasmids of pcDNA and XBP1s for 48 h. **g** Activation of ER stress in the liver of age-matched male mice fed an HFD for 2–32 weeks. Right panel, densitometric analysis of XBP1s in the liver (*n* = 4). **h** The mRNA levels of ERdj4, p58^ipk^, CHOP, and ATF6 in the liver of age-matched mice fed a regular diet or HFD (18 weeks). Each bar represents the mean ± s.e.m. *, *P* < 0.05, Student’s *t*-test. **c**, **e**, **g** Each lane represents a mouse sample

As we found that LPS induced both P300 and ER stress (Fig. 2c, d, i), we tested whether P300 induction is through LPS-mediated activation of ER stress. We found that ER stress triggered by thapsigargin or tunicamycin, an agent widely used to model cellular ER stress, led to the P300 induction in Hepa1-6 cells, with ER stress occurring before P300 induction (Fig. 3a and Supplementary Fig. 2a). Alleviation of ER stress by the chemical chaperone TUDCA, previously shown to protect against ER stress^15^, blocked thapsigargin-induced P300 in hepatocytes (Fig. 3b). In addition, treatment with TUDCA-negated hepatic P300 protein induction by HFD feeding (Fig. 3c). We next determined the pathways leading to P300 induction in the ER stress response. Depletion of IRE1 by adenoviral shRNA abolished P300 induction by LPS; in comparison, depletion of either PERK or ATF6 had no effect on LPS-mediated P300 induction (Supplementary Fig. 2b–d**)**. In the ER stress response, activated IRE1 leads to excision of an intron from the immature XBP1 transcript, resulting in the replacement of the C-terminal domain of the XBP1 protein and generation of the active XBP1s^27^. We found that depletion of XBP1 by adenoviral shRNA abolished LPS-mediated P300 induction in Hepa1-6 cells (Fig. 3d), and P300 could not be induced by an HFD in liver-specific XBP1 knockout mice^28^ (Fig. 3e and Supplementary Fig. 2e). On the other hand, overexpression of XBP1s increased P300 in a concentration-dependent manner (Fig. 3f and Supplementary Fig. 2f). These data demonstrate that activation of the IRE1-XBP1s pathway leads to P300 induction. Moreover, in agreement with previous reports on obese animals and human subjects^29–32^, HFD feeding resulted in augmented XBP1s protein levels (Supplementary Fig. 2g). Furthermore, prolonged HFD feeding also led to an increase in hepatic XBP1s protein levels (Fig. 3g) and the mRNA levels of XBP1s’s downstream target gene, such as ERdj4 and p58^ipk^ (Fig. 3h). The activation of the IRE1-XBP1 pathway was temporally associated with a marked increase in P300 protein levels as shown previously (Fig. 1d and Supplementary Fig. 2g).

**Fig. 4:**
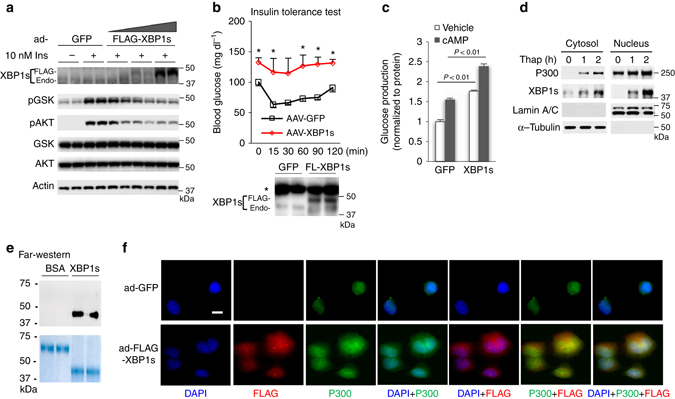
Overexpression of XBP1s impairs insulin signaling and increases the cytoplasmic localization of P300. **a** Forty-eight hours after the addition of adenoviral-GFP and-FLAG-tagged XBP1s, Hepa1-6 cells were subjected to 4 h serum starvation and treated with insulin (10 nM) for 10 min. **b** WT mice were injected with AAV-TBG-GFP and AAV-TBG-XBP1s (3 × 10^11^ GC/mouse). An insulin tolerance test (3 h fast, 0.5 unit per kg) was conducted 10 days after viral injection (*n* *=* 4–5). Bottom, expression levels of XBP1s in the liver. Endo, endogenous. *, nonspecific. The indicated significance is the result of a paired sample *t*-test between mice injected with AAV-TBG-GFP and AAV-TBG-XBP1s. **c** Primary hepatocytes were isolated from mice injected with AAV-TBG-GFP and AAV-TBG-XBP1s and subjected to a glucose production assay (0.2 mM cAMP, 4 h) (*n* *=* 3). **d** Hepa1-6 cells were treated with 400 nM thapsigargin for indicated time, cytosol and nuclear extracts were prepared. **e** Same amounts of bovine serum albumin and purified FLAG-tagged XBP1s protein were subjected to SDS–polyacrylamide gel electrophoresis (staining with Colloidal blue, *lower*) or transferred to membrane, then incubated with human P300 protein (1 µg) for 16 h, washed, followed by incubation with antibody against P300 (*upper*). **f** 48 h after the addition of adenoviral -GFP and -FLAG-tagged XBP1s, Hepa1-6 cells were subjected to immunofluorescence staining. Scale bar, 10 µm. Data are presented as mean ± s.e.m

Next, we tested whether low-level expression of XBP1s as observed in obese animals and human subjects could affect insulin signaling. We found that the overexpression of FLAG-tagged XBP1s decreased insulin-stimulated AKT and GSK phosphorylation in Hepa1-6 cells (Fig. 4a). Moreover, a threefold more expression of XBP1s than endogenous protein levels in the liver-impaired insulin sensitivity (Fig. 4b). Primary hepatocytes isolated from mice with XBP1s overexpression produced more glucose (Fig. 4c). Our data support a previous study from Shulman’s group that showed that liver-specific XBP1 knockout mice produced significantly less glucose in a hyperinsulinemic-euglycemic clamp experiment, reflecting improved insulin sensitivity after the loss of XBP1^33^. These data suggest that XBP1s is a negative modulator of insulin signaling.

Since HFD feeding also increased P300 protein levels in the cytoplasm of hepatocytes (Fig. 2g–i), and ER stress triggered by thapsigargin increased cytoplasmic and nuclear P300 protein levels (Fig. 4d), we further probed the interaction of XBP1s with P300 by overexpressing FLAG-tagged XBP1s in Hepa1-6 cells. Endogenous P300 was co-immunoprecipitated with XBP1s, and thapsigargin treatment increased the protein levels of XBP1s and P300 as well as the association of P300 with XBP1s (Supplementary Fig. 2h). In addition, endogenous P300 was co-immunoprecipitated with endogenous XBP1s in the liver of *ob/ob* mice (Supplementary Fig. 2i). In a Far-western blot, P300 could bind directly to XBP1s, but not bovine serum albumin (Fig. 4e). In light of the above findings, we asked whether XBP1s could affect cytoplasmic P300 protein levels. To test this possibility, we overexpressed FLAG-tagged-XBP1s in Hepa1-6 cells, and found that more P300 appeared in the cytoplasm in cells with XBP1s overexpression (Fig. 4f and Supplementary Fig. 2j).

### Disruption of insulin signaling by induced hepatic P300 in obesity

**Fig. 5:**
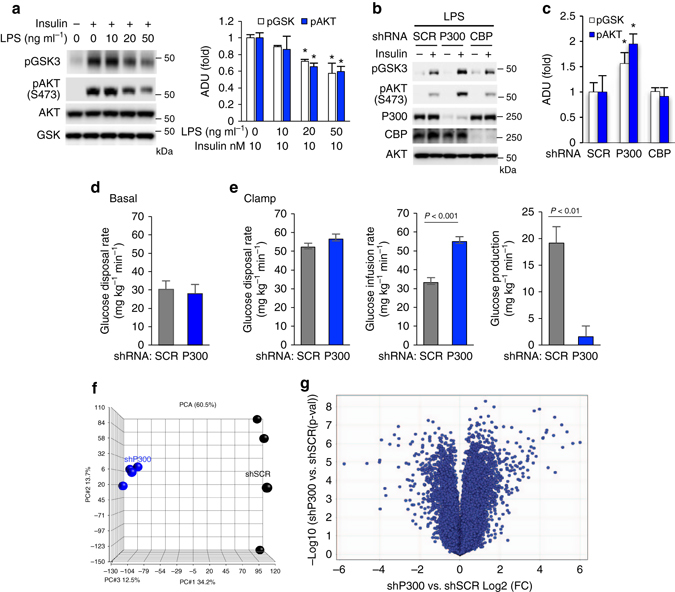
Depletion of P300 improves insulin sensitivity. **a** Hepa1-6 cells were treated with the indicated concentration of LPS for 48 h then insulin (10 nM, 20 min). (*right*) Densitometric analysis of pAKT and pGSK3 in cells treated with insulin (*n* = 3). **b**, **c** Hepa1-6 cells were treated with SCR, CBP and P300 adenoviral shRNA for 24 h, then LPS (500 ng ml^−1^) for 24 h followed by treatment with 10 nM insulin (20 min) **b** and densitometric analysis of pAKT and pGSK3 in cells treated with insulin **c** (*n* = 3). **d**, **e** 48 h after the injection of adenoviral shRNAs, mice were subjected to 5 h fasting before the clamp (*n* = 5/group). Basal glucose production in mice after 5 h fasting **d** and Glucose disposal rates, glucose infusion rates and hepatic glucose production **e** during the clamp experiment. **f** PCA was performed on differentially expressed genes from the liver of mice with SCR or P300 AAV-shRNA injection. **g** Volcano plot is used to analyse differential expression of hepatic genes, and to show the fold change and statistical significance. SCR, scrambled shRNA. Each bar represents the mean ± s.e.m. *, *P* < 0.05, Student’s *t*-test

Since the activation of ER stress can impair insulin signaling^15, 34^ and LPS-induced ER stress (Fig. 2c, d, i), we examined LPS’s effect on insulin signaling in Hepa1-6 cells. LPS treatment significantly decreased the phosphorylation of AKT and GSK3 by insulin, and occurred in a concentration-dependent manner (Fig. 5a and Supplementary Fig. 3a). However, the depletion of P300, but not CBP, significantly enhanced AKT and GSK3 phosphorylation by insulin in LPS-treated cells (Figs 5b, c), revealing the specific role of P300 in the negative regulation of insulin signaling. To test whether P300 had any effect on insulin sensitivity, we used adenoviral shRNA^35^ to deplete P300 in the liver of mice fed an HFD for 2 weeks (Supplementary Fig. 3b). Depletion of hepatic P300 significantly decreased blood glucose levels in a pyruvate tolerance test (Supplementary Fig. 3c). During the hyperinsulinemic-euglycemic clamp experiment, depletion of hepatic P300 did not significantly change the rates of basal glucose disposal (Fig. 5d) and glucose disposal (Fig. 5e, left). The rates of glucose infusion required to sustain euglycemia, however, increased significantly in mice with P300 depletion (Fig. 5e, middle). Depletion of P300 significantly augmented the suppression of HGP by insulin, reflecting an improvement in hepatic insulin sensitivity in HFD-fed mice (Fig. 5e, right). Depletion of P300, but not its closely related-protein CBP, increased insulin-mediated AKT and GSK phosphorylation in hepatocytes (Supplementary Fig. 3d). In comparison, overexpression of P300 decreased AKT and GSK phosphorylation by insulin (Supplementary Fig. 3e). Moreover, principal component analysis demonstrated that the depletion of hepatic P300 (>70%) (Supplementary Fig. 4a, b) by AAV-shRNA markedly changed the transcriptomes in the liver (Fig. 5f). This array analysis identified 827 genes, including genes related to glucose and lipid metabolism, whose hepatic mRNA levels changed by more than twofold (Fig. 5g and Supplementary Fig. 4c–e). Of note, however, the depletion of P300 did not change the mRNA levels of the PTP1b gene.

### Inhibition of P300 activity improves insulin signaling

**Fig. 6:**
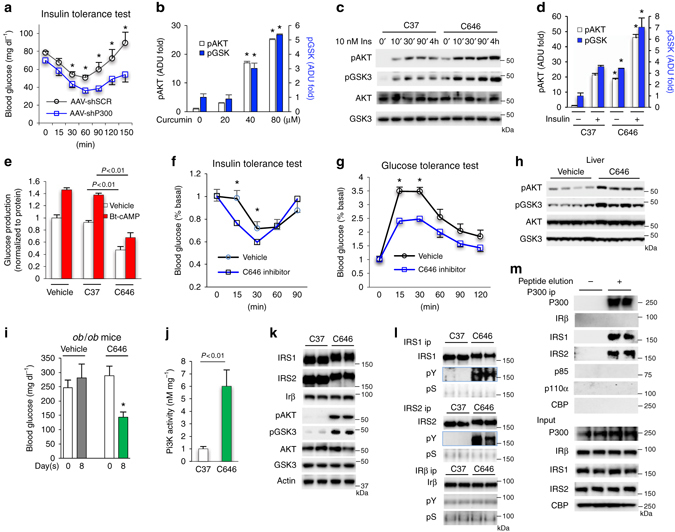
Inhibition of P300 acetyltransferase activity improves insulin signaling. **a** After injection of AAV8-shRNAs for SCR or P300 via jugular vein, mice were fed on an HFD for 3 weeks, insulin tolerance test (6 h fasting, 0.5 unit per kg) was conducted (*n* = 5). **b** Densitometric analysis of the pAKT and pGSK3 in cells treated with curcumin as in Supplementary Fig. 5a. **c**, **d** Hepa1-6 cells were treated with 20 μM control C37 or inhibitor C646 for 1 h before the addition of 10 nM insulin (~4 h). Densitometric analysis of the pAKT and pGSK3 in hepatocytes treated with insulin for 4 h (*n* = 3) **d**. **e** 16 h after the planting of primary hepatocytes, vehicle (DMSO), C37 and C646 (20 µM) were added to medium during serum starvation. After washing with PBS, above agents and 0.2 mM Bt-cAMP were added in glucose production medium (*n* = 3). **f**, **g** 2 weeks after being fed on an HFD, mice were given either the vehicle or inhibitor C646 (15 nmol g^−1^) via intraperitoneal injection for 2 weeks, insulin tolerance test (4 h fasting, 0.6 unit kg^−1^, *n* = 4–5) and glucose tolerance test (4 h fasting, 1.5 g kg^−1^, *n* = 4–5) were conducted. **h** Liver tissues from HFD-fed mice were collected after 16 days of treatment with vehicle or inhibitor C646. Each lane represents a mouse sample. **i** Blood glucose levels (6 h fasting) in *ob/ob* mice treated with vehicle or inhibitor C646 (30 nmol g^−1^) for 8 days (*n* = 5). **j** Hepa1-6 cells were treated with 5 µM C37 or inhibitor C646 for 4 h. Pi3k P110α was immunoprecipitated (*n* = 3). **k**, **l** Hepa1-6 cells were treated with 20 µM C37 or inhibitor C646 for 4 h in DMEM without foetal bovine serum. **m** Liver lysates from mice fed an HFD (3 weeks) were incubated with P300-specific antibody and protein G beads overnight, washed, then incubated with peptide used to generate P300 antibody for 2 h. Each bar represents the mean ± s.e.m. *, *P* < 0.05, paired sample *t*-test between groups

The deacetylase Sirtuin1 has been reported to improve insulin signaling^6–8^, and we have found that the depletion of hepatic P300 by both ad-shRNA and AAV-shRNA improved insulin sensitivity in HFD-fed mice (Figs 5e and 6a). In addition, P300 has intrinsic acetyltransferase activity^36^, so we investigated whether P300 acetyltransferase activity could inhibit insulin signaling. To test this hypothesis, we treated Hepa1-6 cells with curcumin, an inhibitor of CBP/P300 acetyltransferase activity^37^, which increased the phosphorylation of AKT and GSK3 in a concentration-dependent manner (Fig. 6b and Supplementary Fig. 5a). Second, treatment with the P300-acetyltransferase-specific inhibitor C646^38, 39^ increased significantly the phosphorylation levels of AKT and GSK3 both in the absence and presence of insulin when compared to treatment with control inactive compound 37 (Figs 6c, d). In addition, C646 markedly decreased both basal and Bt-cAMP-stimulated glucose production in primary hepatocyte when compared to the treatment with the inactive compound C37 or DMSO vehicle control (Fig. 6e).

To further assess the effect of C646 on improving insulin sensitivity in animals with insulin resistance, HFD-fed mice were given either the vehicle or C646 via intraperitoneal injection for 2 weeks^39^. Treatment with C646 significantly increased insulin sensitivity and glucose tolerance (Fig. 6f, g). Moreover, treatment with C646 drastically decreased the acetylation levels of hepatic IRS1 and IRS2 of *ob/ob* mice along with increased tyrosine phosphorylation of IRS1 and IRS2 and phosphorylation of AKT and GSK in the liver of HFD-fed mice and *ob/ob* mice without the significant changes in body weight (Fig. 6h and Supplementary Fig. 5b–d). Strikingly, administration of C646 in obese *ob/ob* mice for 8 days reduced their high blood glucose levels by 50% (Fig. 6i). Furthermore, treatment with C646 increased Pi3K enzymatic activity by sixfold in Hepa1-6 cells (Fig. 6j), suggesting that the IR or its substrates might be the P300 target sites. Indeed, treatment with C646 caused mobility shifts of both IRS1 and IRS2 (Fig. 6k), which is often associated with phosphorylation of these proteins. Next, we immunoprecipitated ΙRβ as well as IRS1 and IRS2 and then determined tyrosine phosphorylation. C646 significantly increased tyrosine phosphorylation of IRS1 and IRS2 but had a minimal effect on the phosphorylation of IRβ at Y972, the site for IRS recruitment to the IR (Fig. 6l). In addition, both IRS1 and IRS2 were co-immunoprecipitated with P300; however, IRβ was not co-immunoprecipitated with P300 in liver tissues lysates (Fig. 6m). Since HFD feeding induced P300 in the liver, but not in adipose tissues or muscle, and C646 augmented AKT and GSK phosphorylation in the liver, but not in muscle and adipose tissues (Supplementary Fig. 5d–f), we believe that P300 acetyltransferase activity in the liver is the main target site for C646.

### Induced P300 impairs insulin signaling by acetylating IRS1/2

**Fig. 7:**
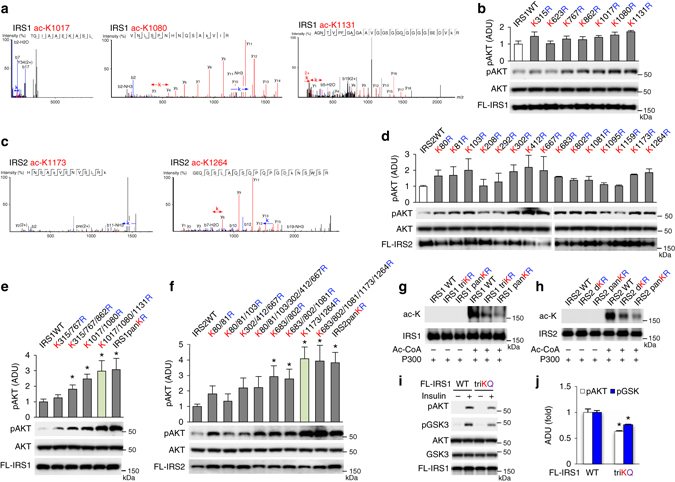
Identification and characterization of acetylation sites in IRS1 and 2. **a**–**d** To mimic the induction of hepatic P300, we overexpressed P300 along with FLAG-tagged-IRS1 or -IRS2 in Hepa1-6 cells, FLAG-tagged-IRS1 and -IRS2 proteins were purified and used to map the acetylation sites by mass spectrometry **a**, **c**. 2 μg of plasmids containing IRS1/2-WT or their mutants were transfected into Hepa1-6 cells, cells were harvested 48 h after transfection (*n* = 3) **b**, **d**. **e**, **f** Plasmids of IRS1 or 2 mutants with combined KR mutations and IRS1 or 2 WT plasmid were transfected into Hepa1-6 cells as above (*n* = 3). *, *P* < 0.05, paired sample *t*-test between groups transfected with WT and mutated IRS plasmids. **g**, **h** Purified Flagged-tagged IRS1/2-WT, and mutant proteins were incubated with 0.2 µg P300 protein for 1 h at 30 °C in the presence and absence of acetyl-CoA. **i**, **j** Forty-eight hours after the transfection of 2 µg IRS1-WT or –triKQ mutated plasmid, Hepa1-6 cells were treated with 10 nM insulin for 10 min **i**. Densitometric analysis of pAKT and pGSK3 in cells treated with insulin (*n* = 3) **j**. Data are presented as mean ± s.e.m

As HFD feeding resulted in cytoplasmic localization of P300 (Fig. 2f, g), and C646 increased the tyrosine phosphorylation of IRS1/2 (Fig. 6l), and IRS1/2 associated with P300 (Fig. 6m), we reasoned that P300 might acetylate cytoplasmic IRS1/2 proteins. Therefore, we used mass spectrometry to map acetylation sites in FLAG-tagged IRS1 and IRS2. We found that lysine residues 315, 623, 767, 862, 1017, 1080 and 1131 in IRS1 and lysine residues 80, 81, 103, 208, 292, 302, 412, 667, 683, 802, 1081, 1095, 1159, 1173 and 1264 in IRS2 could be acetylated. To test the effects of acetylation at these sites upon insulin signaling, we generated IRS1 and IRS2 mutants, in which acetylated lysine residues were substituted with arginine as mimics of non-acetylated lysine (KR mutants). Single mutation (K to R) of these sites in both IRS1 and IRS2 had a mild effect on AKT and GSK phosphorylation (Fig. 7a–d). However, combined KR mutations at 315/767/862, 1017/1080, 1017/1080/1131 lysine residues in IRS1, combined KR mutations at 683/802, 683/802/1081, 1173/1264, 683/802/1081/1173/1264 lysine residues in IRS2 significantly augmented insulin signaling. In particular, IRS1K1017/1080/1131R (triple mutations-triKR) and IRS2K1173/1264R (double mutations-dKR) augmented insulin signaling to a similar degree as their IRS-panKR mutations (Fig. 7e, f and Supplementary Fig. 6a, b). IRS1-triKR mutations and IRS2-dKR mutations dramatically decreased P300-mediated acetylation of IRS in in vitro acetylation assays, and IRS1/2-panKR mutations decreased IRS acetylation by P300 even further (Fig. 7g, h), indicating that the identified acetylation sites in IRS1 and 2 were P300 target sites. Furthermore, transfection with the IRS1-triKQ mutant, in which three lysine residues (IRS1K1017/1080/1131Q) were substituted with glutamine to mimic acetylation of lysine residues, significantly decreased the phosphorylation of AKT and GSK by insulin (Fig. 7i, j).

**Fig. 8:**
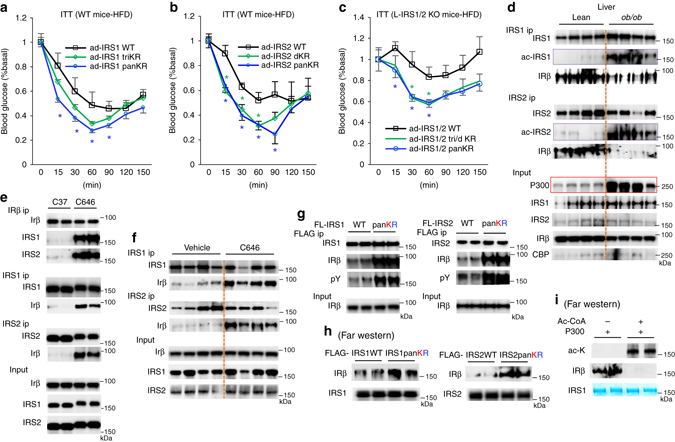
Acetylation of IRS1/2 by P300 decreases their binding to IRβ. **a**, **b** After injection of adenoviral IRS1 and its mutants **a** or adenoviral IRS2 and its mutants **b**, mice were fed an HFD for 2 weeks, then insulin tolerance tests (6 h fasting, 0.5 unit per kg) were conducted (*n* = 5). *, *P* < 0.05, one-way analysis of variance test. **c** After injection of adenoviral IRS1/2-WT, adenoviral IRS1-triKR/IRS2-dKR, or adenoviral IRS1/2 panKR together with AAV8-TBG-Cre, mice were fed an HFD for 16 days, then insulin tolerance tests (6 h fasting, 0.5 unit per kg) were conducted (*n* = 5). **d** Liver tissues were collected from 4-month-old heterozygous (±) lean control and *ob/ob* mice. Immunoprecipitates were immunoblotted with IRS1/2, IRβ, and anti-acetylated lysine antibodies. **e** Hepa1-6 cells were treated with 20 µM C37 or inhibitor C646 for 4 h. **f** Liver tissues from HFD-fed mice were collected after 16 days of treatment with vehicle or inhibitor C646. **g** Forty-eight hours after the transfection of IRS1-WT, IRS1-panKR, IRS2-WT and IRS2-panKR expression plasmids, anti-FLAG magnetic meads were used to pull down these proteins. **h** Same amounts of IRS1-WT, IRS1-panKR or IRS2-WT, IRS2-panKR were employed in a SDS–polyacrylamide gel electrophoresis gel, and transferred onto a membrane, after renaturation, membranes were incubated with IRβ, followed by incubation with anti-IRβ antibody. **i** 36 h after the addition of adenoviral IRS1-WT to overexpress the IRS1 protein, Hepa1-6 cells were treated with inhibitor C646 for 16 h before harvest of cells to block IRS1 acetylation, and this protein was used in the Far-western blot. **d**, **f** Each lane represents a mouse sample. Data are presented as mean ± s.e.m

To confirm the effects of IRS1/2 acetylation on insulin sensitivity in animals, we generated adenoviral IRS1-WT, IRS1-triKR, IRS1-panKR mutant, IRS2-WT, IRS2-dKR, and IRS2-panKR expression vectors. The same amounts of these adenoviruses (1 × 10^10^ GC/mouse) were used to express wild IRS-WT and mutant proteins to comparable amounts as their corresponding endogenous protein levels in the liver of HFD-fed mice. Mice with an injection of adenoviral IRS1-panKR exhibited a significant improvement in insulin sensitivity when compared to mice with an injection of adenoviral IRS1-WT (Fig. 8a). Adenoviral IRS1-triKR injection also improved insulin sensitivity, even though this did not reach statistical significance. In parallel, mice with an injection of either adenoviral IRS2-dKR or IRS2-panKR mutant displayed a significant improvement in insulin sensitivity (Fig. 8b). We further assessed the effects of IRS1/2 KR mutations on insulin signaling in HFD-fed mice with the loss of endogenous IRS1/2 protein. Liver-specific IRS1/2 knockout mice were generated by the injection of AAV8-TBG-Cre into double floxed IRS1/2 mice^40^ (Supplementary Fig. 7a–c). Sixteen days after the injection of IRS1/2-WT and their mutants (1 × 10^10^ GC/IRS/mouse), we performed an insulin tolerance test and found that mice with the injections of adenoviral IRS1-triKR and IRS2-dKR, or IRS1/2-panKR had significantly improved insulin sensitivity when compared to mice with the injections of both adenoviral IRS1-WT and IRS2-WT (Fig. 8c).

### Acetylation of IRS1/2 decreases their association with IRβ

In an obese mouse model (*ob/ob* mouse) with insulin resistance^41^, we found that *ob/ob* mice had significantly higher levels of hepatic P300 protein and IRS1 and 2 acetylation as compared to lean control mice (Fig. 8d). Interestingly, *ob/ob* mice had significantly less IRS1 and 2 associated with IRβ, suggesting that acetylation of IRS1/2 might affect their binding to IRβ. To test this hypothesis, we treated Hepa1-6 cells with C646, and conducted immunoprecipitation assays. C646 drastically increased the association of IRS1 and 2 with IRβ (Fig. 8e). In addition, C646 significantly augmented the association of IRS1 and 2 with IRβ in the liver of HFD-fed mice (Fig. 8f). To further test this hypothesis, we overexpressed FLAG-tagged-IRS1-WT or IRS2-WT to similar levels as their corresponding FLAG-tagged-IRS1-panKR or IRS2-panKR in Hepa1-6 cells. Anti-FLAG antibody was used to pull down IRS1 and 2 and their mutants. We found both IRS1/2-panKR mutants had higher levels of tyrosine phosphorylation as well as associated IRβ when compared to the corresponding FLAG-tagged-IRS1/2-WT (Fig. 8g). To directly prove that the acetylation of IRS1and 2 decreases their binding to IRβ, we conducted Far-western blots in which similar amounts of purified IRS and their mutants were used. After renaturation of these proteins, we tested their bindings to IRβ. Indeed, KR mutation of IRS1 or 2 increased their association with IRβ (Fig. 8h). On the other hand, acetylation of IRS1 by P300 negated its association with IRβ (Fig. 8i).

## Discussion

Increased LPS leakage from the gut has emerged as one of the most appealing mechanisms to explain the connections between changes in the intestinal microbiome and insulin resistance^15, 18, 19, 42^, but the mechanism underlying the impairment of hepatic insulin signaling is not well understood. We found that LPS can induce P300, which occurs through the activation of the IRE1-XBP1 pathway. P300 could not be induced by LPS in hepatocytes after the depletion of XBP1, and HFD feeding was unable to induce hepatic P300 in liver-specific XBP1 knockout mice. These data indicate that XBP1 plays an important role in P300 induction. Indeed, overexpression of XBP1s as a model to bypass the activation of the IRE1-XBP1 pathway increased P300 protein levels, and importantly, overexpression of XBP1s could change the cellular localization of P300. P300 is mainly a nuclear protein; however, significant amounts of P300 appeared in the cytoplasm in cells with the activation of ER stress and with XBP1s overexpression. HFD feeding led to the activation of the IRE1-XBP1 pathway and the generation of XBP1s. Subsequently, XBP1s could increase the cytoplasmic levels of P300. P300 protein has a relatively fast turnover rate and a 5 h half-life^35^, suggesting that continuous synthesis of P300 in the cytoplasm is required for maintaining P300 protein levels. Since XBP1s can bind directly to P300, this interaction might prevent the transport of P300 from the cytoplasm into the nucleus as well as the degradation of P300, thus, augmenting cytoplasmic P300 protein levels. However, the interaction domains of P300 and XBP1s and their importance in relocating P300 into the cytoplasm still need to be elucidated.

P300 is associated with IRS1 and 2 in cellular extracts from the liver of HFD-fed mice, and the inhibition of P300 acetyltransferase activity increased tyrosine phosphorylation of IRS1/2, but not IRβ. Furthermore, increased acetylation levels of IRS1 and 2 in the liver of *ob/ob* mice were accompanied by decreased association of IRS1 and 2 with IRβ, and inhibition of P300 acetyltransferase activity increased the association of IRβ with IRS1 and 2, these results suggest that acetylation of IRS1 and 2 by P300 hinders their association with IRβ. This subsequently impairs the tyrosine phosphorylation by IRβ because IRβ is the kinase for the tyrosine phosphorylation of IRS1/2^43, 44^. This notion was tested first in hepatocytes via overexpression of IRS1/2-WT and IRS1/2-panKR mutants. We found higher levels of association of IRβ with IRS1/2-panKR mutants than with IRS1/2-WT. Second, in a Far-western blot, more IRβ was bound to IRS1/2-panKR mutants. Third, we used P300 to acetylate IRS1 protein that was purified from Hepa1-6 cells treated with C646 to block the acetylation and then conducted another Far-western blot. Acetylation of IRS1 abolished IRβ binding to IRS1 protein. Interestingly, C646 treatment increased the tyrosine phosphorylation of IRS, without changing the serine phosphorylation levels (Figs 6k, i), suggesting that the impairments of insulin signaling by acetylation and the serine phosphorylation of IRS have different mechanisms. It is also possible that the acetylation of IRS1/2 might facilitate the dephosphorylation of tyrosine residues in IRS1/2, therefore disrupting the insulin signaling.

Our data showed that P300 is a prime factor leading to the development of hepatic insulin resistance by acetylating IRS1/2 in the early stages of obesity. Therefore, P300 acetyltransferase activity is a therapeutic target. Indeed, inhibition of P300 acetyltransferase activity improved insulin signaling in cultured hepatocytes and in the liver. Particularly, C646 improved insulin sensitivity in mice fed an HFD and significantly ameliorated hyperglycemia in *ob/ob* mice, making C646 is a potential agent for the treatment of obesity and T2D. Moreover, C646 increased AKT and GSK phosphorylation in the absence of insulin, suggesting that P300 acetyltransferase inhibitors may offer promise for the treatment of type 1 diabetes.

A previous report showed that liver-specific XBP1 knockout mice exhibited increased insulin sensitivity in a hyperinsulinemic-euglycemic clamp experiment. Importantly, the loss of hepatic XBP1 led to increased tyrosine phosphorylation of both IRS1 and IRS2^33^. Since HFD feeding resulted in a moderate ~3-fold increase in XBP1s protein levels, we expressed threefold more XBP1s than endogenous protein levels and found that threefold overexpression of XBP1s impaired insulin sensitivity. Our data support the notion that XBP1s is a negative modulator of insulin signaling^33^. Furthermore, we found that augmented XBP1s leads to P300 induction and cytoplasmic localization and subsequently, IRS1 and IRS2 acetylation and the impairment of insulin signaling. These results offer an explanation for increased tyrosine phosphorylation of both IRS1 and IRS2 in liver-specific XBP1 knockout mice^33^. Of note, the overexpression of XBP1s in the liver has been documented to improve insulin sensitivity^45^. In this report, high levels of XBP1s overexpression were required in order to affect insulin sensitivity^45^. However, there is only a 2–3-fold increase in XBP1s mRNA and protein levels in the liver of obese patients with insulin resistance and in HFD-fed mice^29–32^. Thus, such high levels of XBP1s overexpression in the liver^45^ are unreachable under ER stress and physiologically irrelevant.

## Methods

### General experimental approaches

No samples were excluded from the reported analyses. Samples were not randomized to experimental groups. Animal experiments were not conducted in a blinded fashion.

### Antibodies and immunoblots

A detailed description of the antibodies used in this work is presented in Supplementary Table 1. The original images of immunoblots are shown in Supplementary Figs 8–22.

### Plasmids, adenoviruses and adeno-associated viruses (AAV)

The BLOCK-iT adenoviral RNAi expression system (Invitrogen) was used to construct adenoviral shRNAs for P300 and scrambled shRNA vectors as we previously described^46^. Subsequently, these vectors were employed to generate AAV-vectors. Regions in the pENTR/U6 vector containing the U6 promoter, Pol III terminator and P300shRNA oligo or scrambled shRNA oligo were amplified by PCR and cloned into the AAV-BASIC vector (Vector Biolabs); these vectors were used to make AAV8 shRNAs for P300 and scrambled shRNA. The mouse IRS1 and 2 genes were gifts from Ronald Kahn (Addgene plasmid #11026, #11372)^47^, and these IRS1 and 2 genes were used to generate FLAG-tagged IRS1 and 2. IRS1 and 2 mutants were created using site-directed mutagenesis (Stratagene)^48^. FLAG-tagged IRS1-WT, -triKR/-panKR mutants, -triKQ mutant and IRS2-WT, -dKR/-panKR mutants were subcloned into the pENTR2B vector (Invitrogen), and transferred into the pAd/CMV/V5-DEST vector (Invitrogen) by recombination to generate adenoviral expression clones.

### Cell cultures and transfection

Lipofectamine 2000 (Invitrogen) was used to transfect plasmids into mouse Hepa1-6 cells (ATCC CRL-1830). After 48 h of transfection, cells were harvested or exposed to 10 nM insulin for 10 min before being harvested.

### Glucose production assay and acetylation assay

Mouse primary hepatocytes were cultured in William’s medium E supplemented with ITS (BD Biosciences) and dexamethasone. After 16 h of planting, cells were washed with PBS twice, and the medium was changed to foetal bovine-free DMEM supplemented with vehicle (DMSO), 20 µM C37, or C646. After 3 h of serum starvation, cells were washed twice with PBS, and the 1 ml glucose production medium was supplemented with vehicle (DMSO), 20 µM C37, or C646, and Bt-cAMP. After 3 h incubation with glucose production medium, both the medium and cells were collected. The medium was used to determine glucose concentrations with EnzyChrom Glucose Assay Kit^39^. To acetylate IRS1 and 2, 2 µg of IRS1 or 2 was added to the reaction containing 50 mM Tris-HCl, pH 8.0, 5% glycerol, 0.1 mM EDTA, 50 mM KCl, 1 mM dithiothreitol (DTT), 1 mM PMSF, 10 mM sodium butyrate, 0.2 µg acetyl-CoA and 0.2 µg P300 (Active Motif). Samples were incubated at 30 °C for 1 h.

### Euglycemic hyperinsulinemic clamp experiment

Three-month-old mice were used in this study. Male mice were randomly divided into two groups, and mice were fed on an HFD for 2 weeks. At feeding time day 10, surgical catheterization of the jugular vein with PE-10 tubing was performed. After 3 days of recovery, 1 × 10^9^ PFU of adenovirus was infused through catheter tubing; then, 48 h after viral infusion, mice were subjected to 5 h fasting before the clamp procedure as previously described^23, 35^. Regular human insulin was infused at 5 mU kg^−1^ min^−1^, and glucose levels were measured every 5 min. An infusion of 30% glucose was adjusted to maintain blood glucose at 100–120 mg dl^−1^.

### Measurement of hepatic endotoxin levels and Pi3K activity

Liver tissues were homogenized in an ice-cold water bath and centrifuged for 10 min at 2500 *g* to remove connective tissue. The Limulus Amebocyte Lysate Endotoxin Detection Assay kit (Lonza, Walkersville, MD) was used to determine hepatic endotoxin levels following the steps recommended by the manufacturer. For the measurement of Pi3K enzymatic activity, Hepa1-6 cells were treated with 5 µM C37 or C646 inhibitor for 4 h. P110α antibody (Cell Signaling) was used to pull down Pi3K, and its enzymatic activity was measured using the PI3K-Kinase Activity ELISA: Pico kit (Echelon Biosciences).

### Animal experiments

All animal protocols were approved by the Institutional Animal Care and Use Committee of the Johns Hopkins University. Male C57BL/6 mice, CD14 mice, and *ob/ob* mice were purchased from the Jackson Laboratory. AAV8-scrambled shRNA or P300shRNA (1 × 10^13^ GC/mouse) was injected into mice through the jugular vein. To test C646’s effect on insulin sensitivity in HFD-fed mice, C57BL/6 mice were fed an HFD (60% calories from fat) for 2 weeks starting at the age of 8 weeks. Mice were given either vehicle or C646 (15 nM g^−1^, ordered from Sigma) via intraperitoneal injection for 2 weeks^39^. For the treatment of *ob/ob* mice with C646, 4-month-old *ob/ob* mice were randomly divided into two groups, for which vehicle or C646 (30 nM/ g^−1^) was given through intraperitoneal injection. To test the effect of IRS1/2 mutants on insulin sensitivity, 3-month-old C57BL/6 mice were injected with each adenovirus through the jugular vein (1 × 10^10^ GC/mouse); 2 days after the viral injection, mice were fed on an HFD. After 2 weeks of HFD feeding, an insulin tolerance test was conducted. To generate liver-specific XBP1 and double IRS1 and 2 knockout mice, 1 × 10^11^ GC/mouse of AAV8-TBG-Cre (Vector Core at University of Pennsylvania) was injected through the jugular vein. LPS (0.5 µg g^−1^ body weight/day) was administrated to C57BL/6 mice through intraperitoneal injection for 2 weeks. For treatment with TUDCA, we followed the same protocol for the administration of TUDCA as previously described^49^. Mice were randomly divided into three groups; one group of mice was fed on a regular diet, while the other two groups of mice were fed on an HFD and treated with either vehicle (water) or TUDCA (250 µg g^−1^ body weight, twice a day at 08:00 and 20:00 hours) through intraperitoneal injection for 2 weeks. For tolerance tests, mice were injected intraperitoneally (ip) with 0.5–0.8 unit per kg insulin, 1.5 g kg^−1^ glucose and 1.5 g kg^−1^ pyruvate after fasting for 4–6 h. The primers used for the measurement of mouse endogenous IRS1 by flanking 5'-UTR were as follows: 5'-primer (5'-GGCATGAAACGCCCTTAAAC-3') and 3'-primer (5'-GGAGGAAGCTCGCAGAAAT-3'). The primers used for the measurement of mouse endogenous IRS2 by flanking 3'-UTR were as follows: 5'-primer (5'-CGGGCAGAGAGACCTGAA-3') and 3'-primer (5'-CGCTGTGGTTGTTGTTGTTG-3').

### Immunoprecipitation and preparation of cytoplasmic and nuclear extracts

Cellular lysates were passed 15 times through a syringe needle to break cells on ice. P300, ΙRβ, and IRS1 and 2 were immunoprecipitated using P300 antibody (Santa Cruz), ΙRβ antibody (Cell Signaling), and IRS1 and 2 antibodies (Millipore). The reaction was incubated at 4 °C for 16 h, followed by the addition of protein G beads (Active Motif) to pull down the target protein and its associated proteins. Cytoplasmic and nuclear extracts were prepared using the CelLytic NuCLEAR Extraction kit (Sigma).

### Microarray analysis

Three-month-old C57BL/6 mice were injected with AAV8-scrambled shRNA or P300shRNA (1 × 10^13^ GC/mouse) through the jugular vein. One week after the viral injection, mice were fed an HFD for another week before being killed after 6 h fasting. Liver tissues were harvested. Microarray analysis was conducted in the Johns Hopkins Deep Sequencing and Microarray Core Facility.

### Far-western blot

A far-western blot was conducted using a modified method of Wu et al.^50^ Purified IRS1/2-WT protein or their mutants were separated using a NuPAGE™ Novex™ Tris-Acetate Protein Gel (Thermo Fisher Scientific) and transferred onto a polyvinylidene fluoride membrane. The membrane was incubated in AC buffer (100 mM NaCl, 20 mM Tris (pH 7.6), 0.5 mM EDTA, 10% glycerol, 0.1% Tween-20, 2% skim milk powder and 1 mM DTT) with 6 M guanidine–HCl for 30 min at room temperature. After washing, the membrane was blocked with 5% milk in PBS-Tween (PBST) buffer (4 mM KH_2_PO_4_, 16 mM Na_2_HPO_4_, 115 mM NaCl (pH 7.4), and 0.05% Tween-20) for 1 h at room temperature, and then, the membrane was incubated in protein-binding buffer (100 mM NaCl, 20 mM Tris (pH 7.6), 0.5 mM EDTA, 10% glycerol, 0.1% Tween-20, 2% skim milk powder and 1 mM DTT, prepared freshly) plus 5 µg of ΙRβ (16 h, 4 °C). After three washes with PBST buffer, the membrane was incubated with anti-IRβ (Cell Signaling) overnight at 4 °C.

### Mapping acetylation sites in IRS1 and 2

The FLAG-tagged IRS1 or 2 expression vector was co-transfected with the P300 expression vector^35^ into Hepa1-6 cells. FLAG-tagged-IRS1 or 2 protein was purified and used to map acetylation sites by mass spectrometry in the Johns Hopkins Mass Spectrometry and Proteomics Facility.

### Statistical analyses

Statistical significance was calculated with a Student’s *t*-test and analysis of variance test. Significance was accepted at the level of *P* < 0.05. Sample size (number of mice) was determined on the basis of our previous studies^23, 35^. At least three samples per group were chosen for statistical meaningful interpretation of results and differences in the studies using the Student’s *t*-test and analysis of variation.

### Data availability

Data that support the findings of this study have been deposited in GEO with accession codes: GSE86108. All other data are available from the authors upon reasonable request.

## Electronic supplementary material

Supplementary Information
