# Supplementary material for: Endotoxemia-mediated activation of acetyltransferase P300 impairs insulin signaling in obesity
Source: Nat Commun. 2017 Jul 25;8:131. doi: 10.1038/s41467-017-00163-w (PMC5526866; doi:10.1038/s41467-017-00163-w)
Supplement: Supplementary file 1 — Supplementary Information [file 41467_2017_163_MOESM1_ESM.pdf]

File name: Supplementary Information
Description: Supplementary figures and supplementary table.

Supplementary Table 1 **Antibodies used for immunoblots**

Name of Antibody	Manufacturer Catalog#	Species	Dilution used
**P300**	Santa Cruz, sc-585<br>Abcam, ab14984	Rabbit<br>Mouse	1:200<br>1 ug/ml
**CBP**	Santa Cruz, sc-369	Rabbit	1:200
**CRTC2**	Cell Signaling, 13017	Mouse	1:1000
**FoxO1**	Cell Signaling, 9454	Rabbit	1:1000
**CREB**	Cell Signaling, 9104	Mouse	1:1000
**pPERK**	Cell Signaling, 3179	Rabbit	1:1000
**pIRE1**	Abcam, ab48187	Rabbit	1:1000
**IRE1**	Abcam, ab37073	Rabbit	1 ug/ml
**β-Actin**	Santa Cruz, sc-81178	Mouse	1:5000
**Tubulin**	Cell Signaling, 2144	Rabbit	1:1000
**Lamin A/C**	Cell Signaling, 4777	Mouse	1:2000
**XBP1**	Santa Cruz, sc-7160	Rabbit	1:500
**pGSK3**	Cell Signaling, 9331	Rabbit	1:1000
**pAKT (473)**	Cell Signaling, 9271	Rabbit	1:1000
**GSK3**	Cell Signaling, 9315	Rabbit	1:1000
**AKT**	Abcam, ab8805	Rabbit	1:1000
**IRS1**	Cell Signaling, 3407	Rabbit	1:1000
**IRS2**	Millipore, MABS15	Mouse	0.5 ug/ml
**IRb**	Cell Signaling, 3025	Rabbit	1:1000
**p85**	Cell Signaling, 4292	Rabbit	1:1000
**P110α**	Cell Signaling, 4255	Rabbit	1:1000
**phospho-tyrosine**	Millipore, 05-321	Mouse	1 ug/ml
**phospho-serine**	Millipore, AB1603	Rabbit	1:500
**Anti-Acetylated Lysine**	Cell Signaling, 9814	Rabbit	1:1000
**Anti-FLAG**	Sigma, F3165	Mouse	2 ug/ml
**Anti-HA**	Cell Signaling, 3724	Rabbit	1:1000
**Anti-ubiquitin**	Cell Signaling, 3933	Rabbit	1:1000
**Anti-phospho-IR (972)**	Millipore, 07-838	Rabbit	1:1000

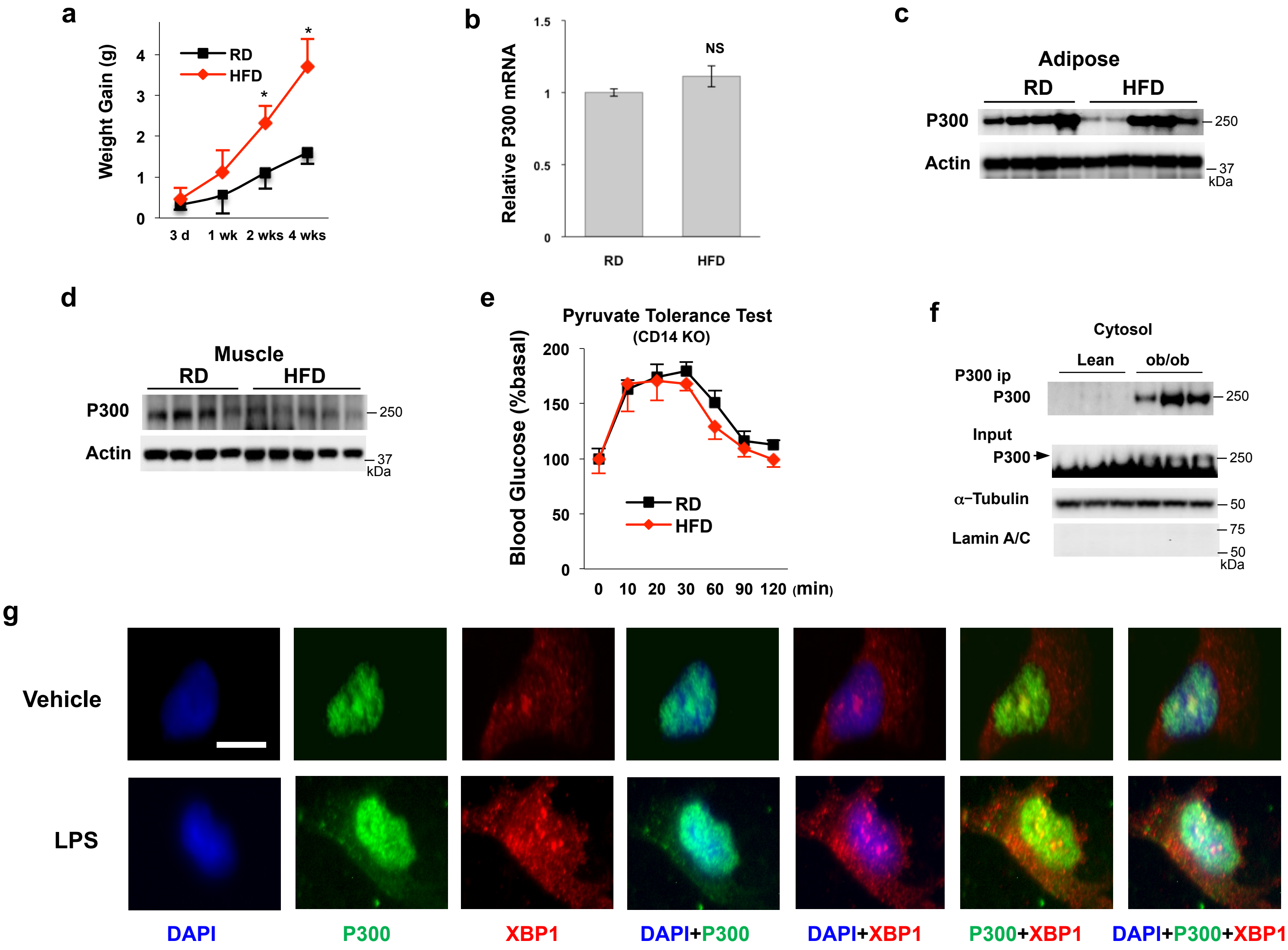

Supplementary Figure 1

Supplementary Figure 1 LPS treatment changes the cellular localization of P300 in hepatocytes. (**a**) Body weight gain in mice fed a regular chow diet or a HFD for 1~4 weeks (*n=4/group*). *, $p<0.05$, paired sample t-test between groups at each time point. (**b**) Hepatic mRNA levels of P300 from mice fed a regular chow diet or a HFD for 2 weeks (normalized to 36B4 expression levels, *n=4*). (**c, d**) P300 protein levels in adipose tissues (**c**) and muscle (**d**) from mice fed a regular chow diet or a HFD for 2 weeks (*n=4~5/group*). (**e**) Pyruvate tolerance test (4 h fasting, 2 g/kg) in CD14 knockout mice fed a HFD for 2 weeks (*n=4/group*). (**f**) Cytoplasmic P300 proteins from lean control and *ob/ob* mice were immuneprecipitated using P300 antibody (Santa Cruz) and immuoblotted with P300 antibody (abcam). Each lane represents a mouse sample. (**g**) 48 h after the treatment with LPS (50 ng/ml), Hepa1-6 cells were subjected to immunofluorescence staining. Scale bar, 10 μm.

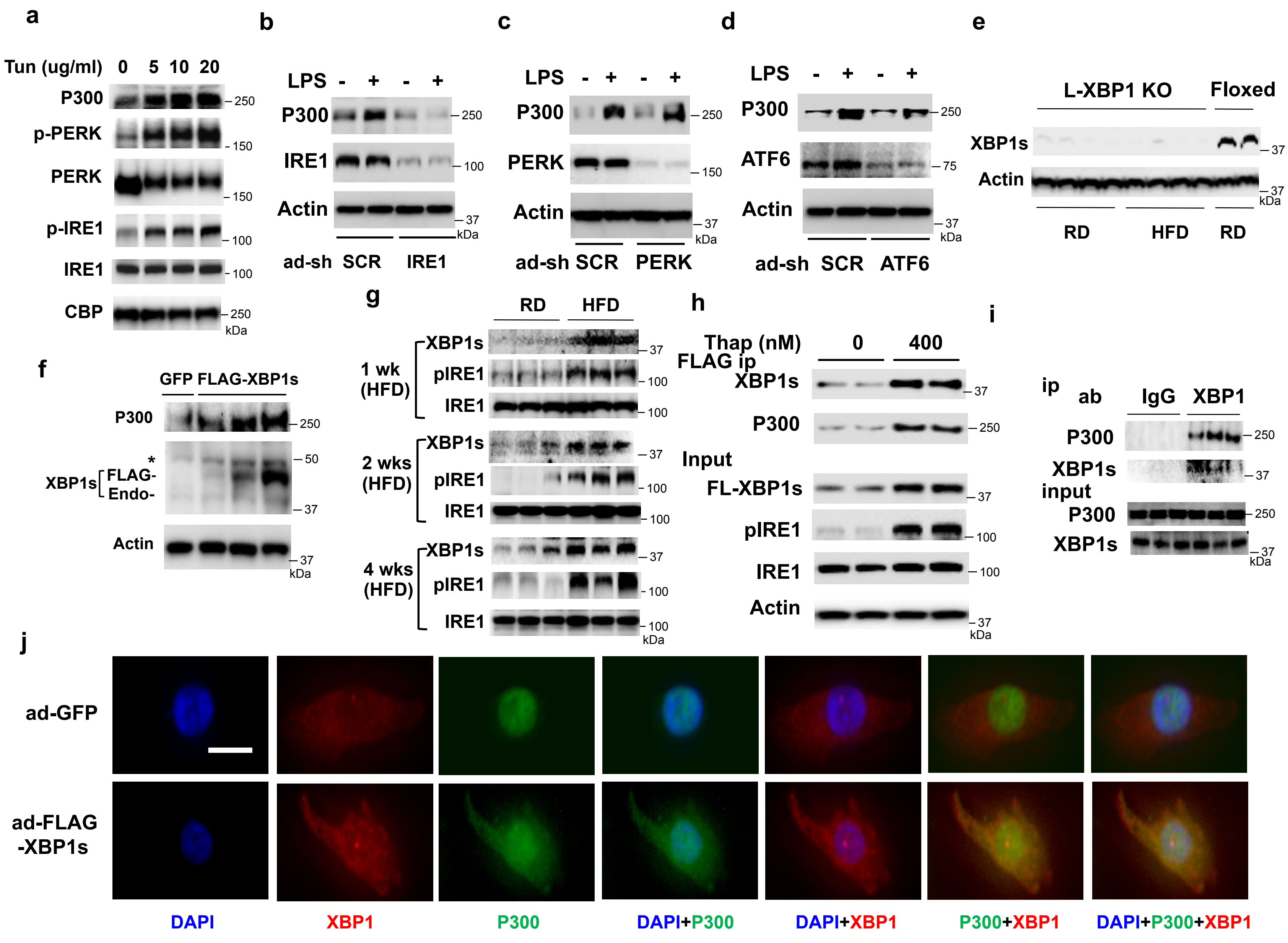

Supplementary Figure 2

Supplementary Figure 2 Activation of IRE1-XBP1 pathway induces P300 in hepatocytes. (**a**) Hepa1-6 cells were treated with indicated amounts of tunicamycin for 12 h. (**b-d**) 24 h after the addition of SCR, IRE1, PERK or ATF6 adenoviral shRNAs, Hepa1-6 cells were treated with LPS for another 24 h. (**e**) Homozygous floxed XBP1 mice were injected with AAV8-TBG-Cre (1 x 10^{11} GC/mouse) through the jugular vein, and fed on a HFD for 2 weeks. Each lane represents a mouse sample. (**f**) Immunoblot of lysates from Hepa1-6 cells transfected with AAV-GFP or AAV-XBP1s for 5 days. (**g**) Activation of ER stress in the liver of mice fed an HFD for 1, 2, and 4 weeks. (**h**) Hepa1-6 cells were infected with FLAG-tagged XBP1s and treated with thapsigargin (400 nM, 6 h). FLAG-tagged XBP1s proteins were immunoprecipitated with anti-FLAG M2 magnetic beads (Sigma). (**i**) Liver lysates from *ob/ob* mice (16 weeks) were incubated with XBP1 specific antibody and protein G beads overnight, washed, and immunoblotted with P300 and XBP1 antibodies. Each lane represents a mouse sample. (**j**) 48 h after the addition of adenoviral -GFP and –FLAG-tagged XBP1s, Hepa1-6 cells were subjected to immunofluorescence staining. Scale bar, 10 µm.

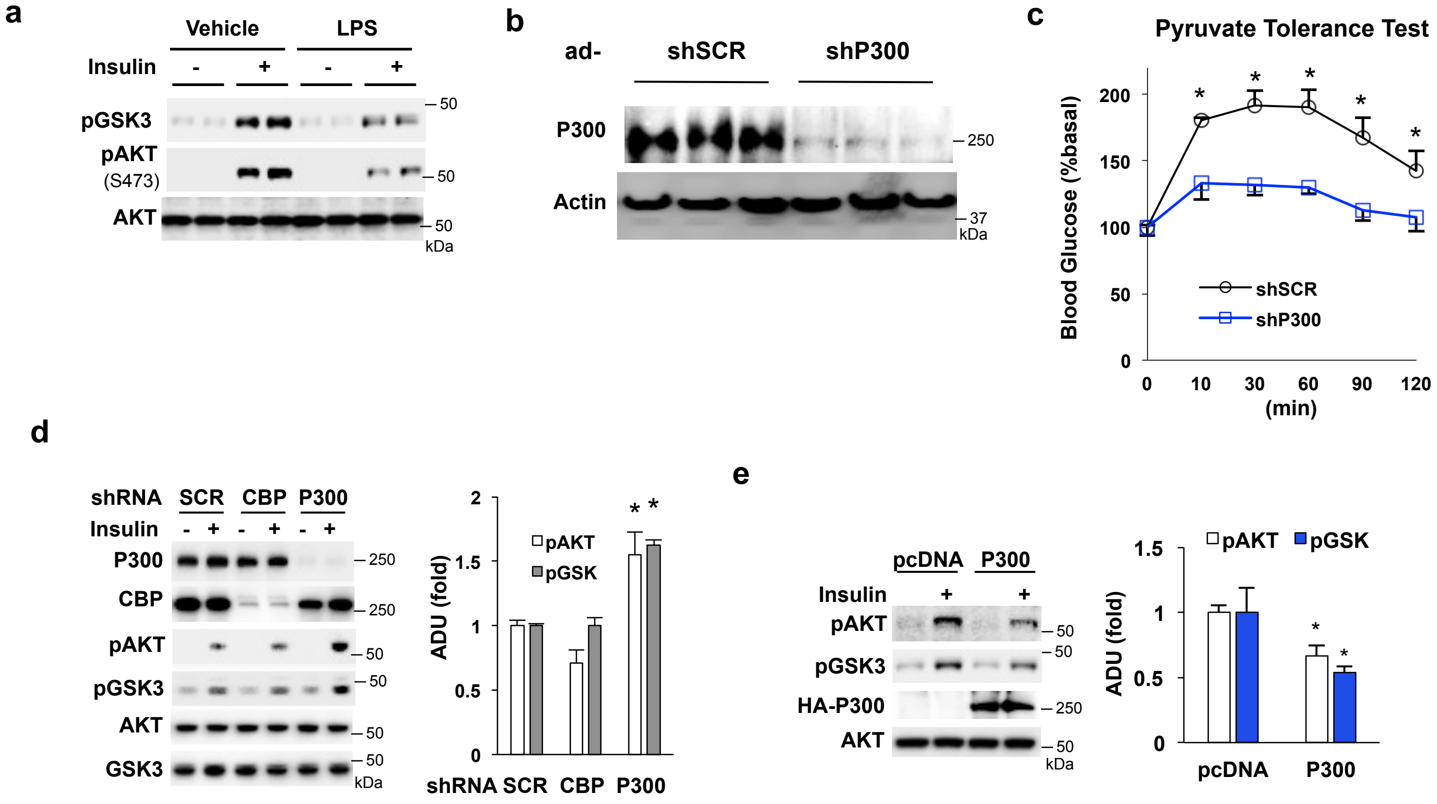

Supplementary Figure 3

Supplementary Figure 3 P300 impairs insulin signaling. (**a**) Hepa1-6 cells were treated with LPS (500 ng/ml) overnight, then insulin (10 nM). (**b**) Liver tissues were collected 48 h after the injection of ad-shRNAs. (**c**) Mice were injected with the ad-shRNAs of SCR or P300 after 2 weeks feeding of a RD diet or HFD (*n=4*). (**d**) Hepa1-6 cells were treated with adenoviral shRNAs of SCR, CBP, and P300 for 48 h, followed by 2 h serum starvation, and then treatment with 10 nM insulin for 20 min (left). Right, densitometric analysis of the pAKT and pGSK3 in cells treated with insulin (*n=3/group*). (**e**) 48 h after the transfection of pcDNA or P300 expression plasmid, Hepa1-6 cells were subjected to 2 h serum starvation, followed by treatment with 10 nM insulin for 10 min. Right, Densitometric analysis of the pGSK3 and pAKT in hepatocytes treated with insulin (*n=3/group*). *, $p<0.05$, paired sample t-test between groups.

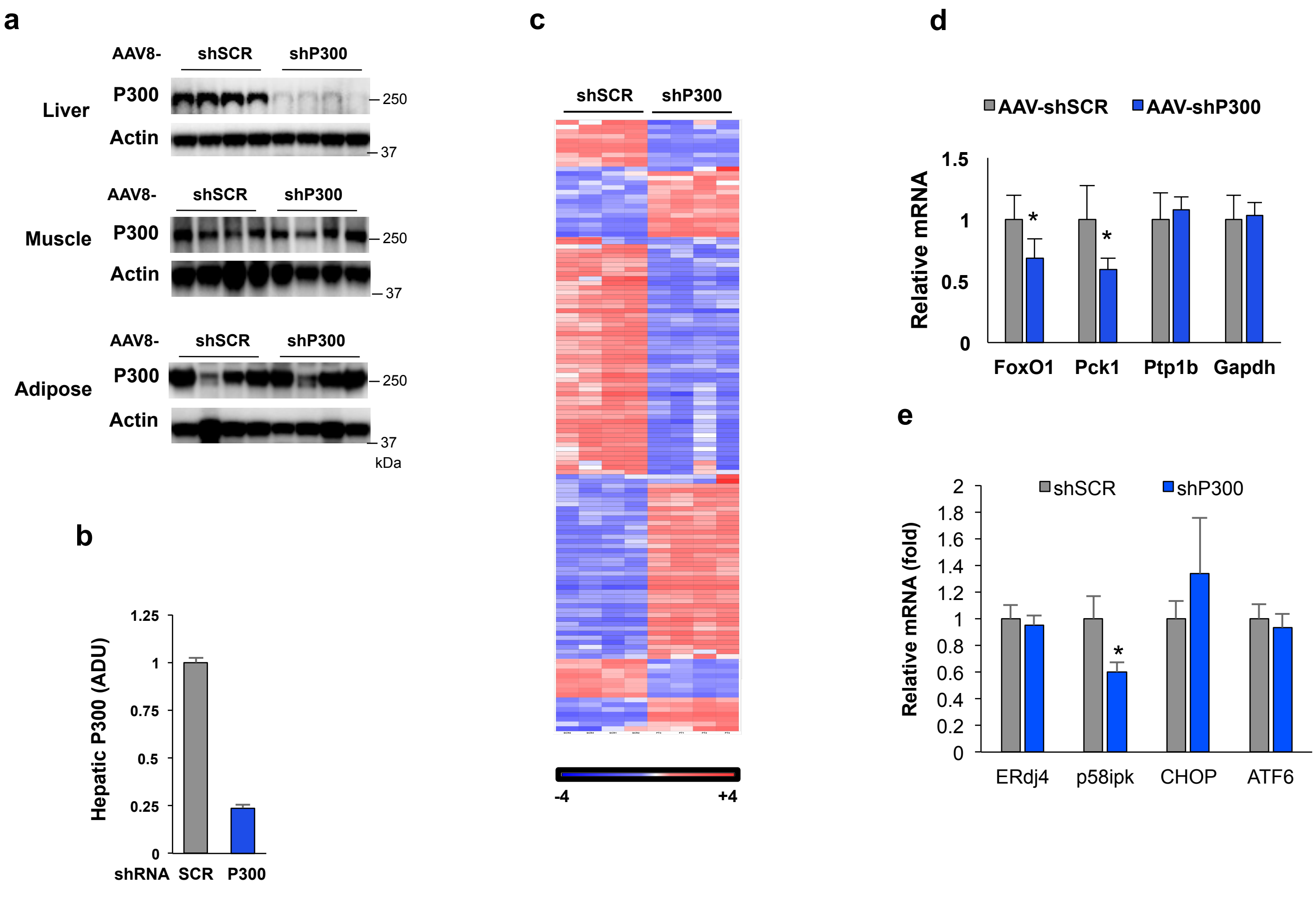

Supplementary Figure 4

Supplementary Figure 4 Depletion of P300 by AAV8-shRNA alters the gene expression in the liver. (**a,b**) After the injection of AAV8-shRNAs for SCR or P300 via jugular vein, mice were fed on a HFD for 2 weeks. P300 protein levels were depleted in the liver, not in muscle or adipose tissues (**a**). Densitometric analysis of P300 protein levels in the liver of mice injected with AAV8-shRNAs for SCR or P300 (*n=4/group*) (**b**). (**c,d**) Depletion of hepatic P300 altered the expression of genes related to glucose and lipid metabolism (*n=4/group*). *, $p<0.05$, paired sample t-test between groups. (**e**) The mRNA levels of ERdj4, $p58^{ipk}$, CHOP, and ATF6 in the liver of mice treated as in (**c**) (*n=4*). (**a,c**) Each lane represents a mouse sample.

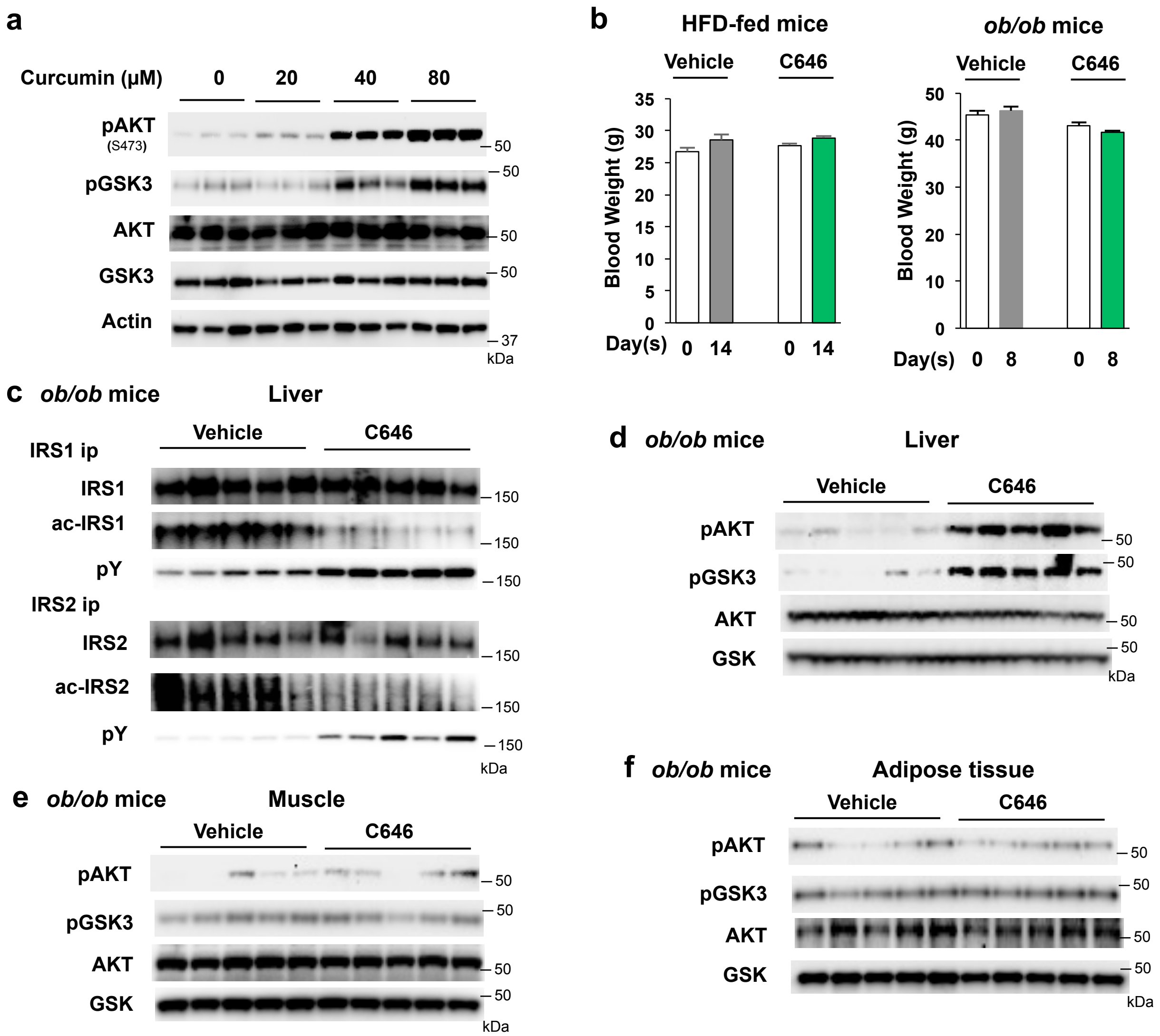

Supplementary Figure 5

Supplementary Figure 5 Inhibition of P300 acetyltransferase activity augments insulin signaling. (**a**) Hepa1-6 cells were treated with indicated amounts of curcumin for 16 h. (**b**) Treatment of HFD-fed mice with vehicle (DMSO) or inhibitor C646 (15 nmol/g) for 14 days (*n=4~5/group*) (**b,** left panel) and *ob/ob* mice with vehicle (DMSO) or inhibitor C646 (30 nmol/g) for 8 days (*n=5/group*) (**b,** right panel). (**c-f**) Immunoprecipitates were immunoblotted with IRS1/2, phospho-tyrosine, and anti-acetylated lysine antibodies (**c**). Inhibitor C646 treatment increased the phosphorylation of AKT and GSK in the liver (**d**), had no effect on the phosphorylation of AKT and GSK in the muscle and adipose tissues (**e,f**). Each bar represents the mean ± s.e.m. Each lane represents a mouse sample.

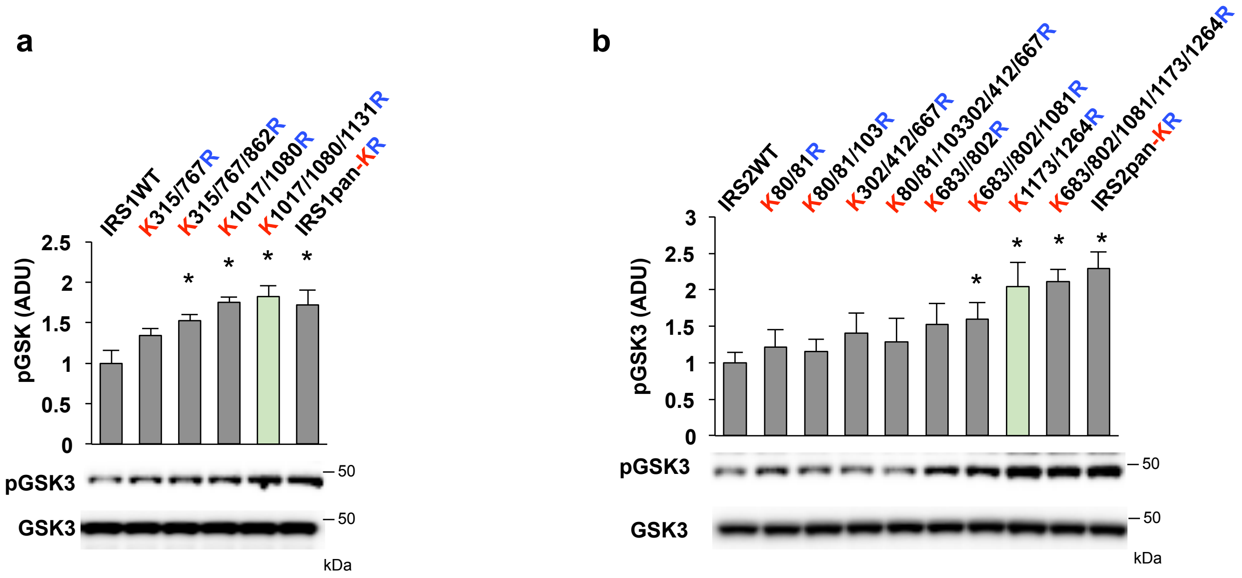

Supplementary Figure 6 Acetylation of IRS1/2 by P300 impairs insulin signaling. (**a,b**) 2ug of plasmids containing IRS1/2-WT or their mutants were transfected into Hepa1-6 cells, cells were harvested 48h after transfection (*n=3*). Each bar represents the mean ± s.e.m. *, $p<0.05$, paired sample t-test between groups transfected with WT and mutated IRS plasmids.

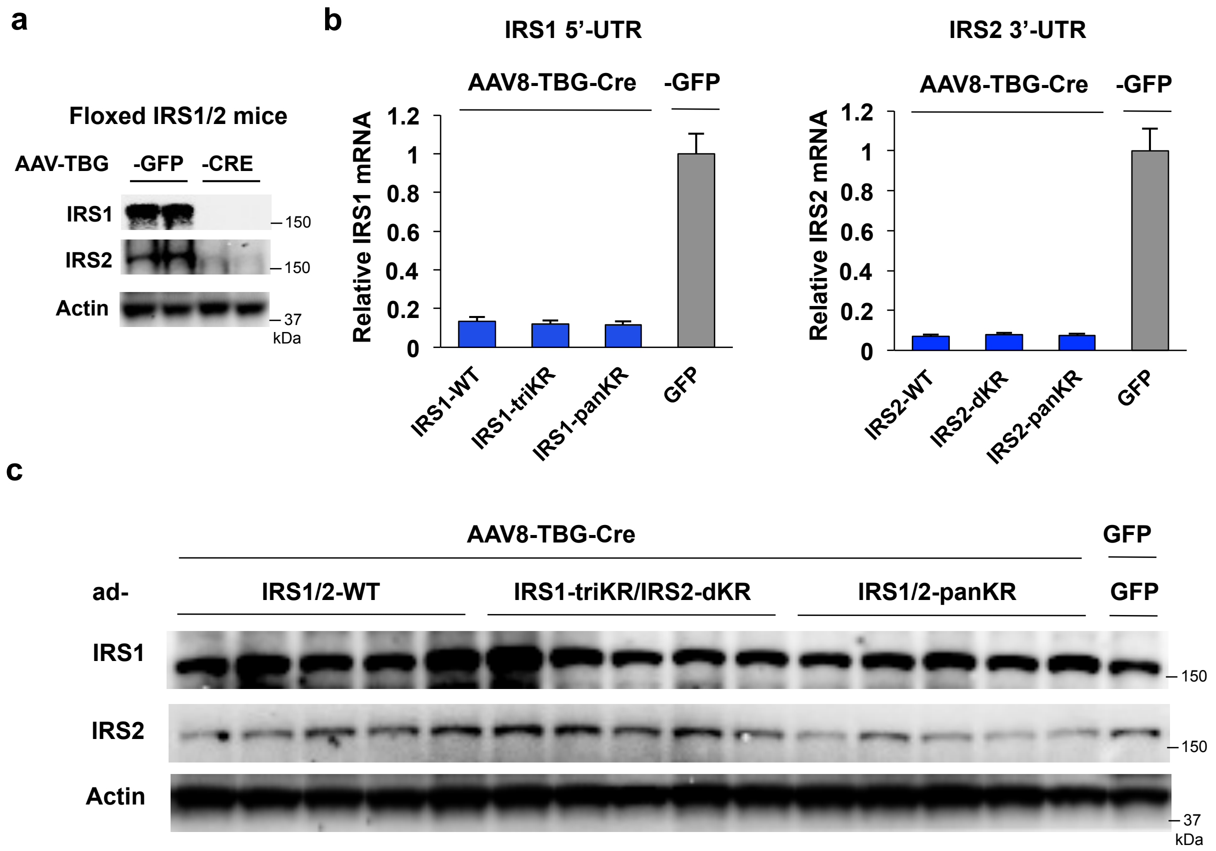

Supplementary Figure 7 Expression of IRS1/2-WT and mutants in liver-specific IRS1/2 knockout mice. (**a**) Homozygous double floxed IRS1/2 mice were injected with AAV8-TBG-Cre (1 x 10^{11} GC/mouse) through the jugular vein, and liver tissues were collected 2 weeks later. (**b,c**) Endogenous IRS1/2 mRNA levels in the liver of mice injected with adenoviral IRS1/2-WT, adenoviral IRS1-triKR/IRS2-dKR, or adenoviral IRS1/2 panKR together with AAV8-TBG-Cre, mice were fed a HFD for 21 days. QPCR primers for endogenous IRS1/2 were designed to flank the untranslated regions of each gene (*n=5/group*) (**b**). Each bar represents the mean ± s.e.m. Protein levels of IRS1 and 2 in the liver (**c**). (**a,c**) Each lane represents a mouse sample.

Fig. 1d

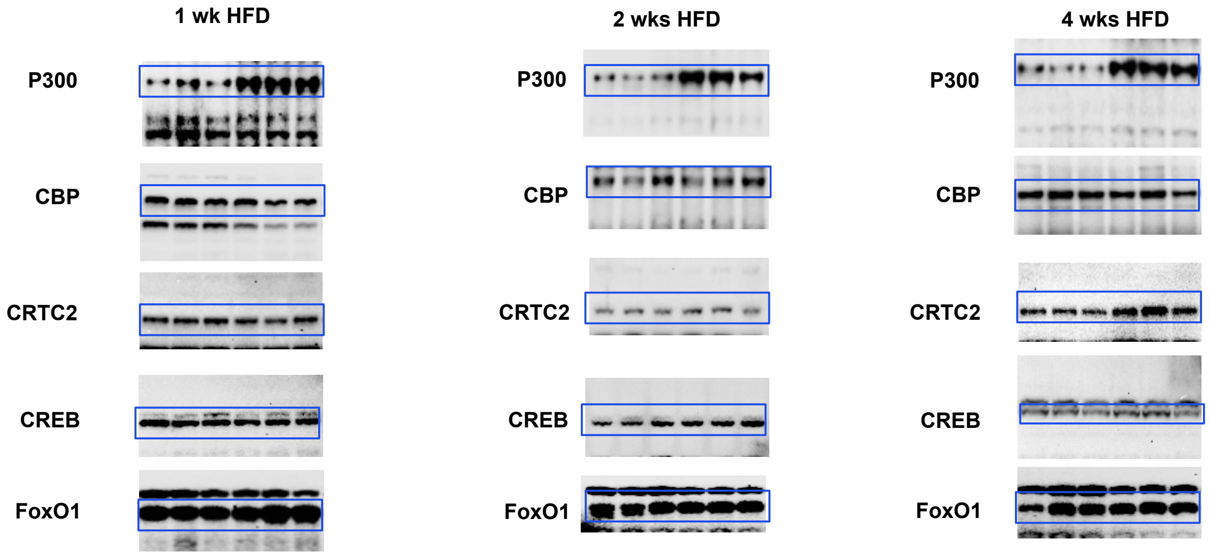

Supplementary Figure 8 Uncropped blots for Fig. 1d. The bands used in the figure are marked in blue squares.

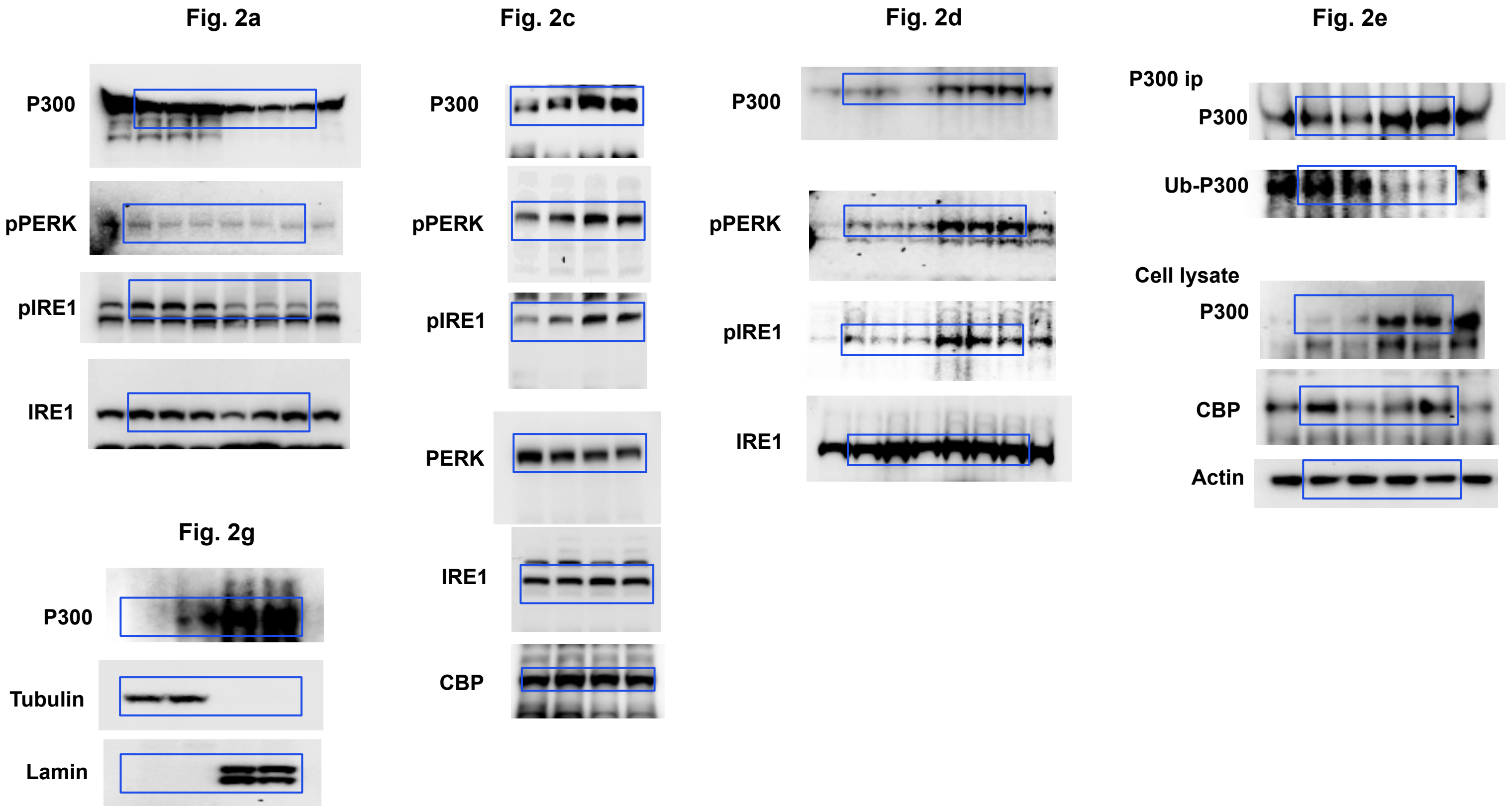

Supplementary Figure 9 Uncropped blots for Fig. 2a, c, d, e, g. The bands used in the figure are marked in blue squares.

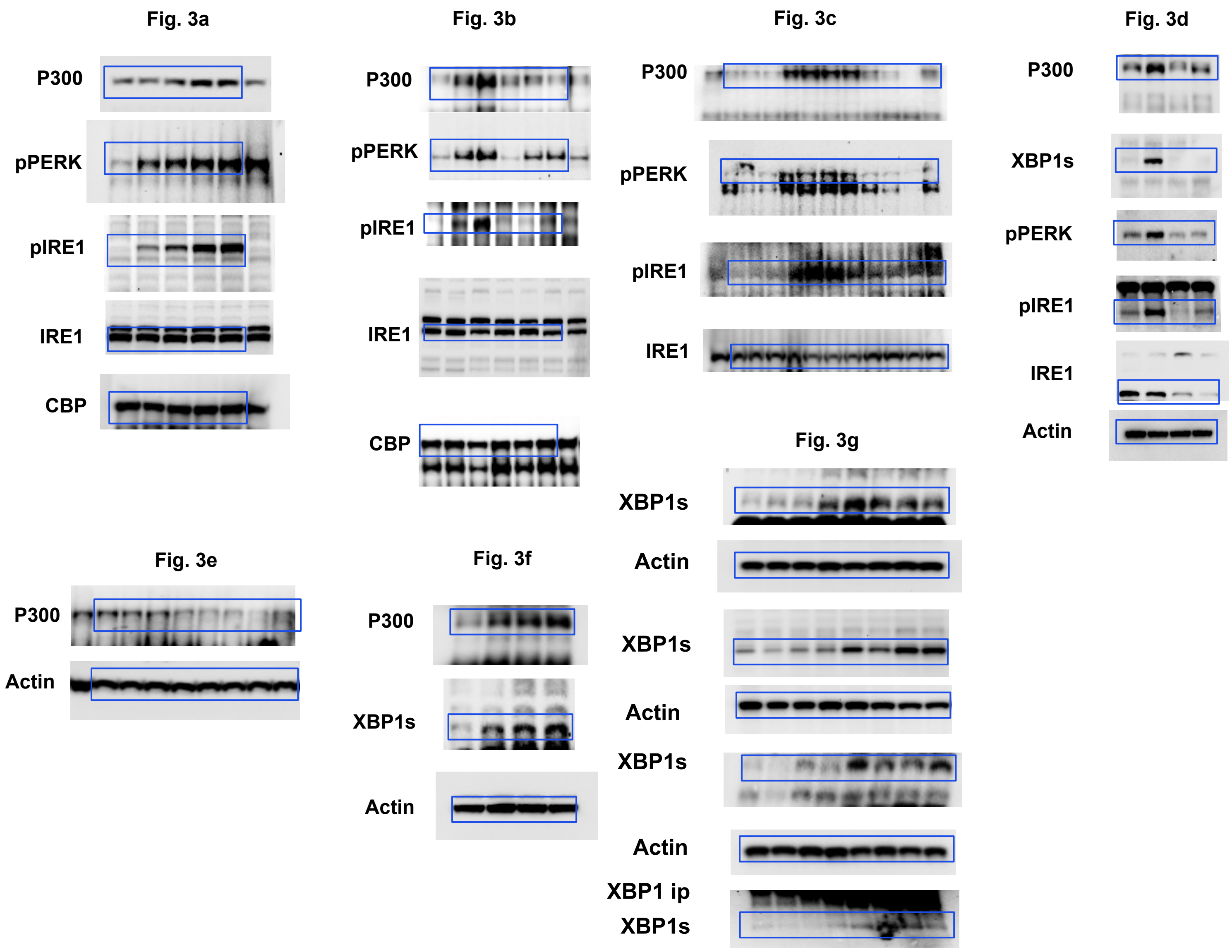

Supplementary Figure 10 Uncropped blots for Fig. 3a-g. The bands used in the figure are marked in blue squares.

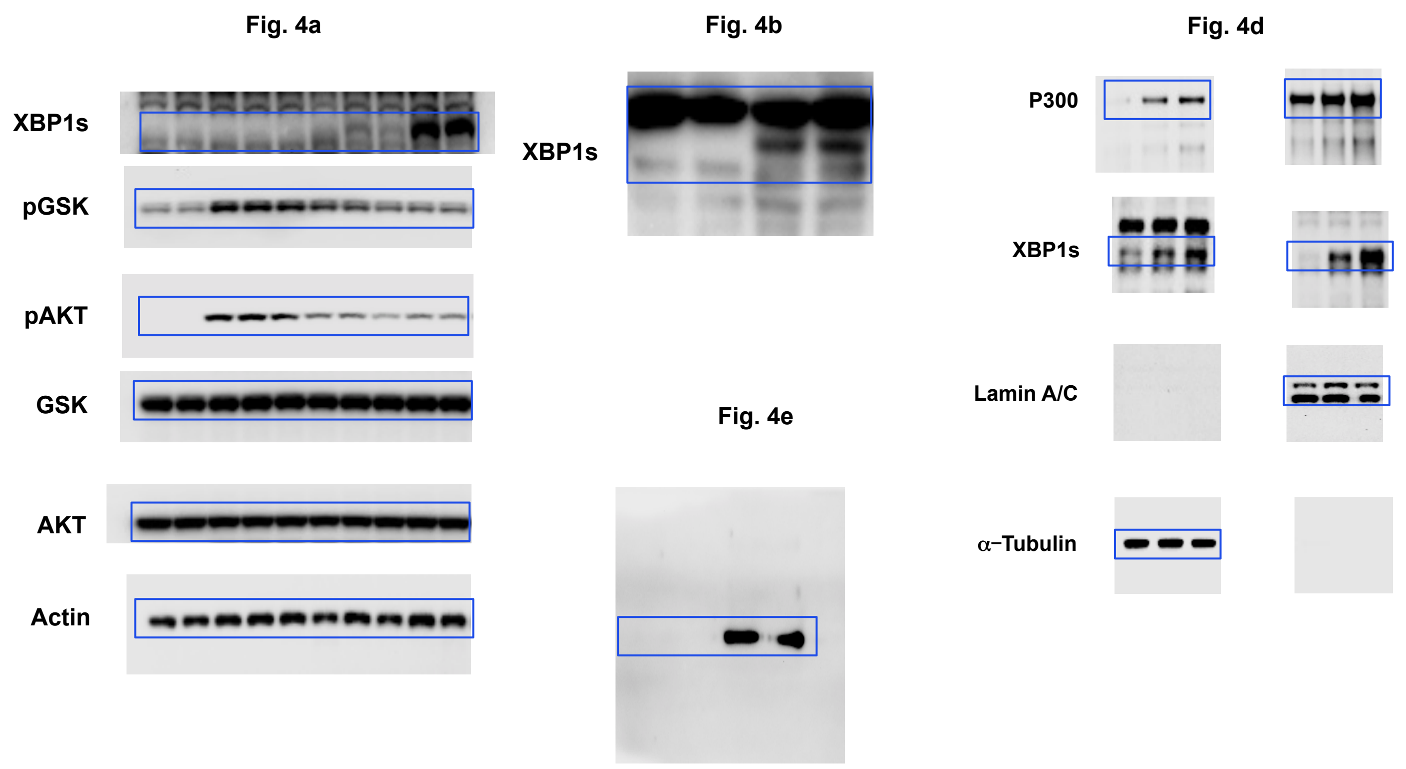

Supplementary Figure 11 Uncropped blots for Fig. 4a, b, d, e. The bands used in the figure are marked in blue squares.

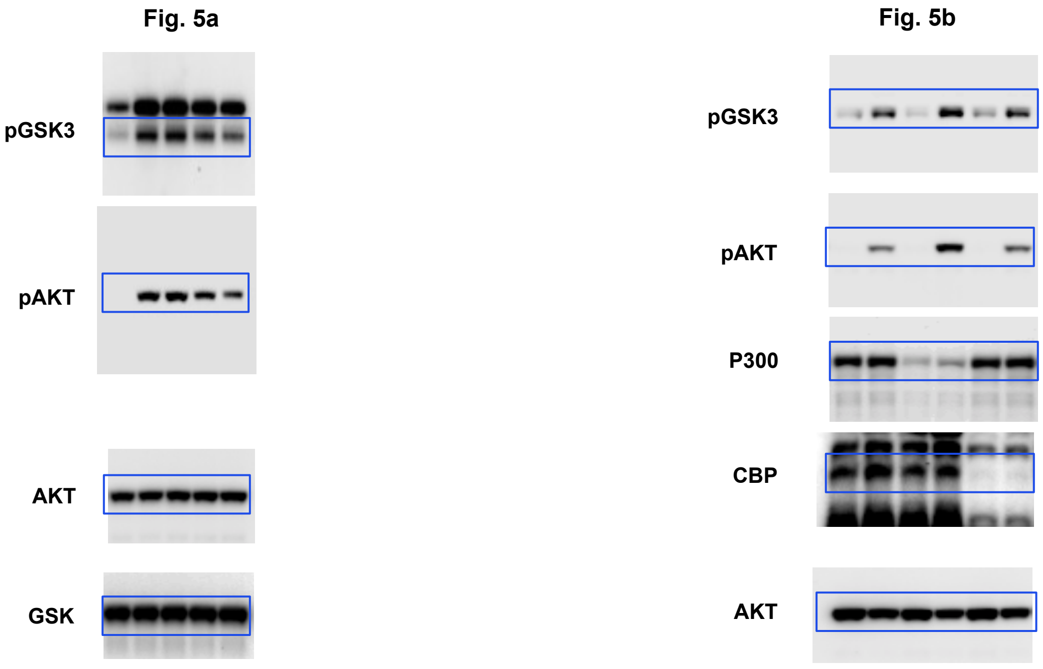

Supplementary Figure 12 Uncropped blots for Fig. 5a, b. The bands used in the figure are marked in blue squares.

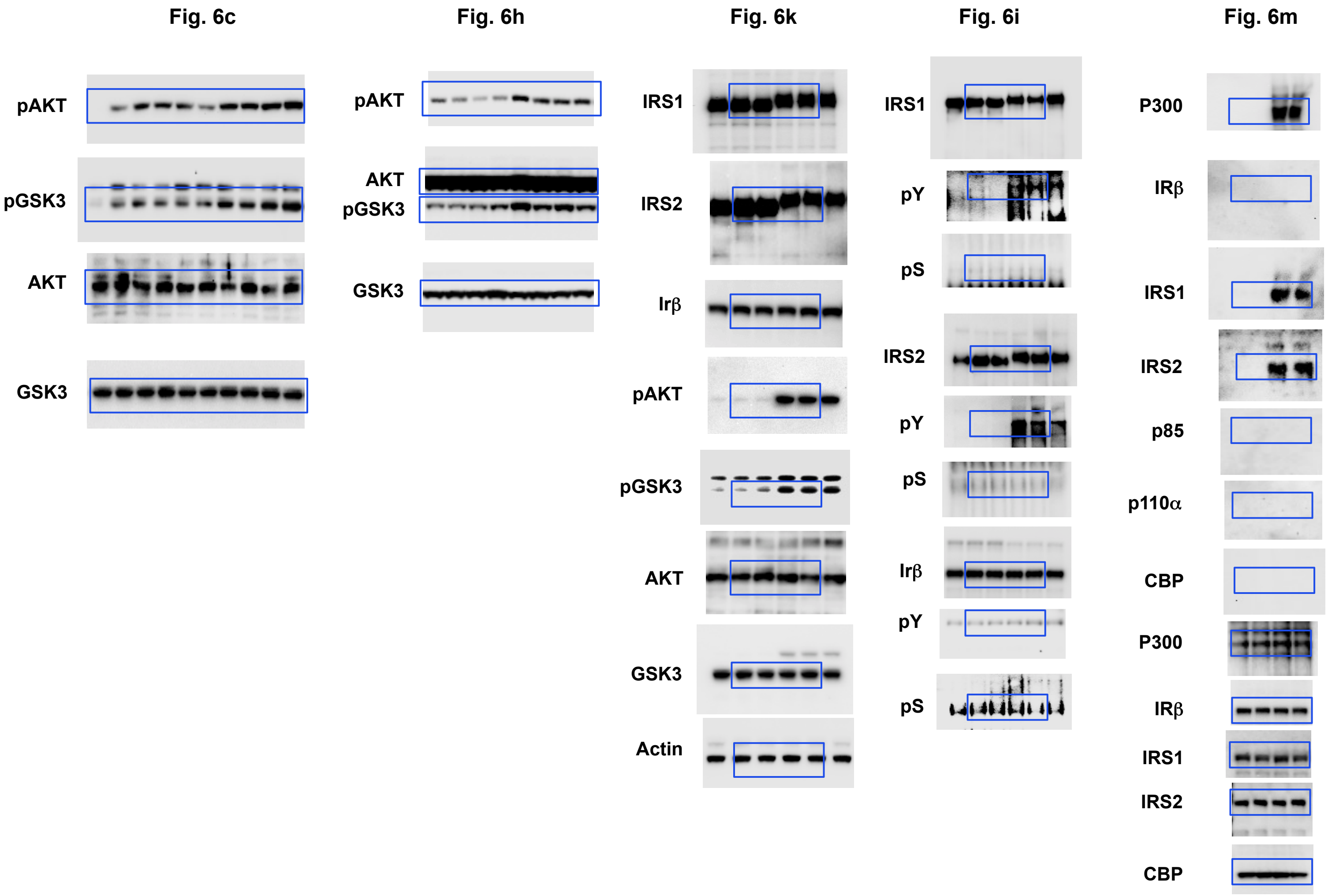

Supplementary Figure 13 Uncropped blots for Fig. 6c, h, k, i, m. The bands used in the figure are marked in blue squares.

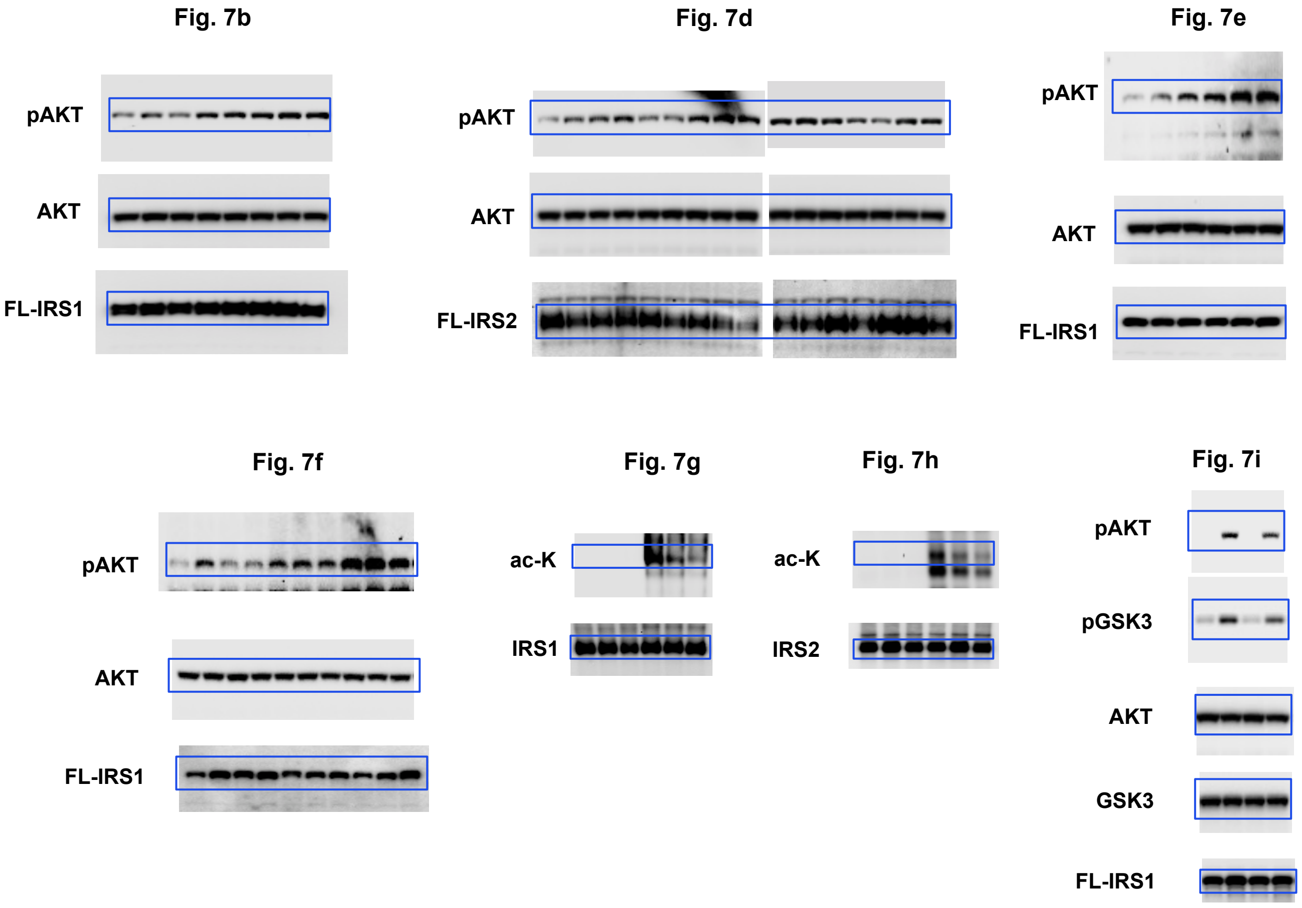

Supplementary Figure 14 Uncropped blots for Fig. 7b, d-i. The bands used in the figure are marked in blue squares.

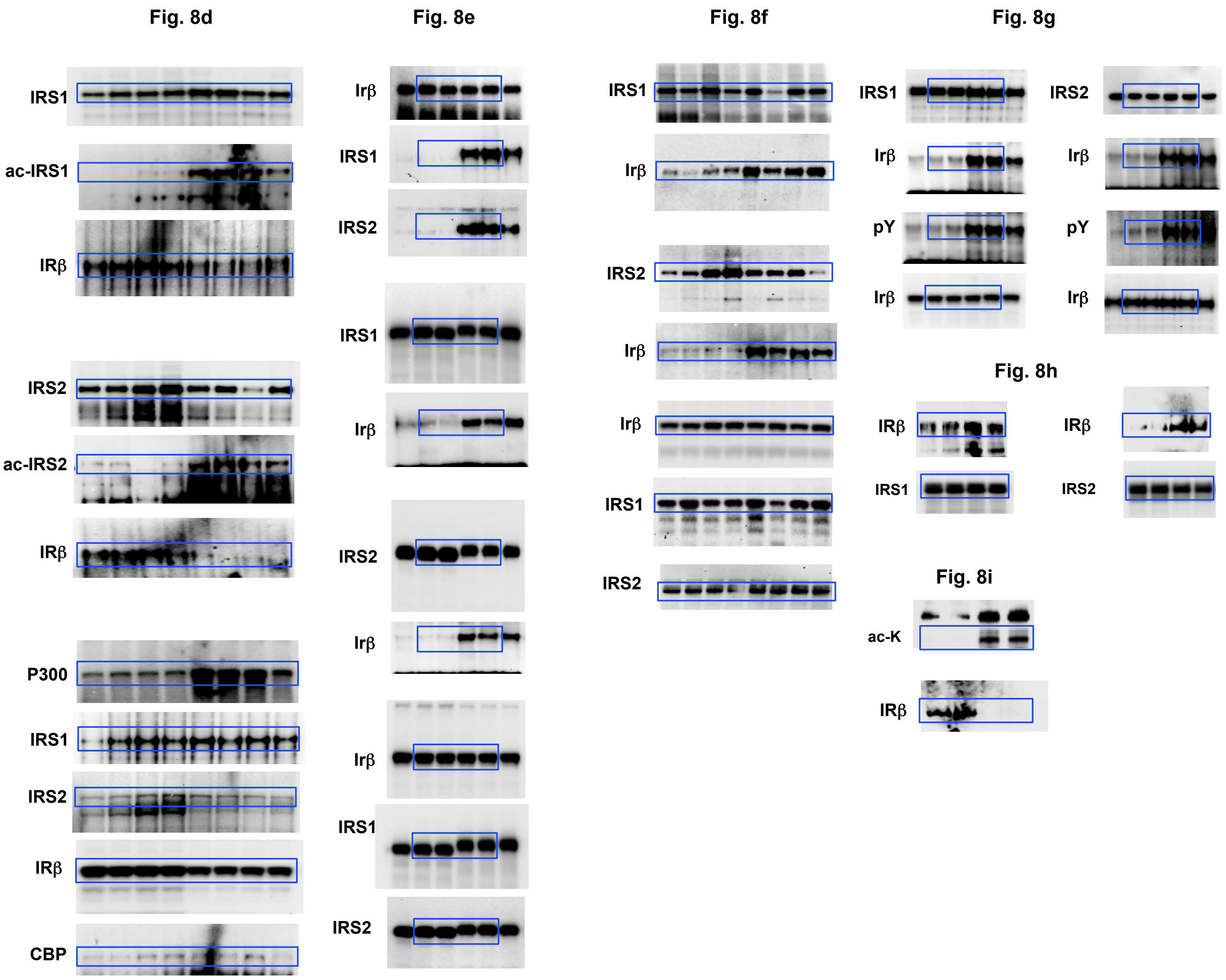

Supplementary Figure 15 Uncropped blots for Fig. 8d-i. The bands used in the figure are marked in blue squares.

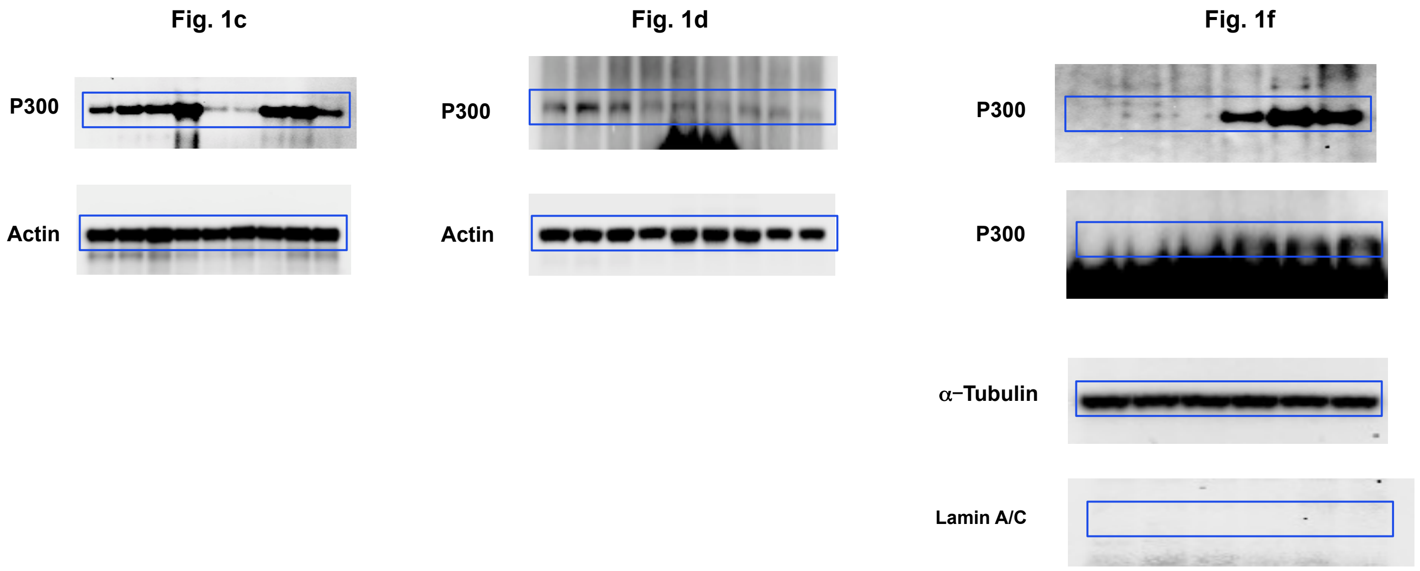

Supplementary Figure 16 Uncropped blots for Supplementary Fig. 1c,d,f. The bands used in the figure are marked in blue squares.

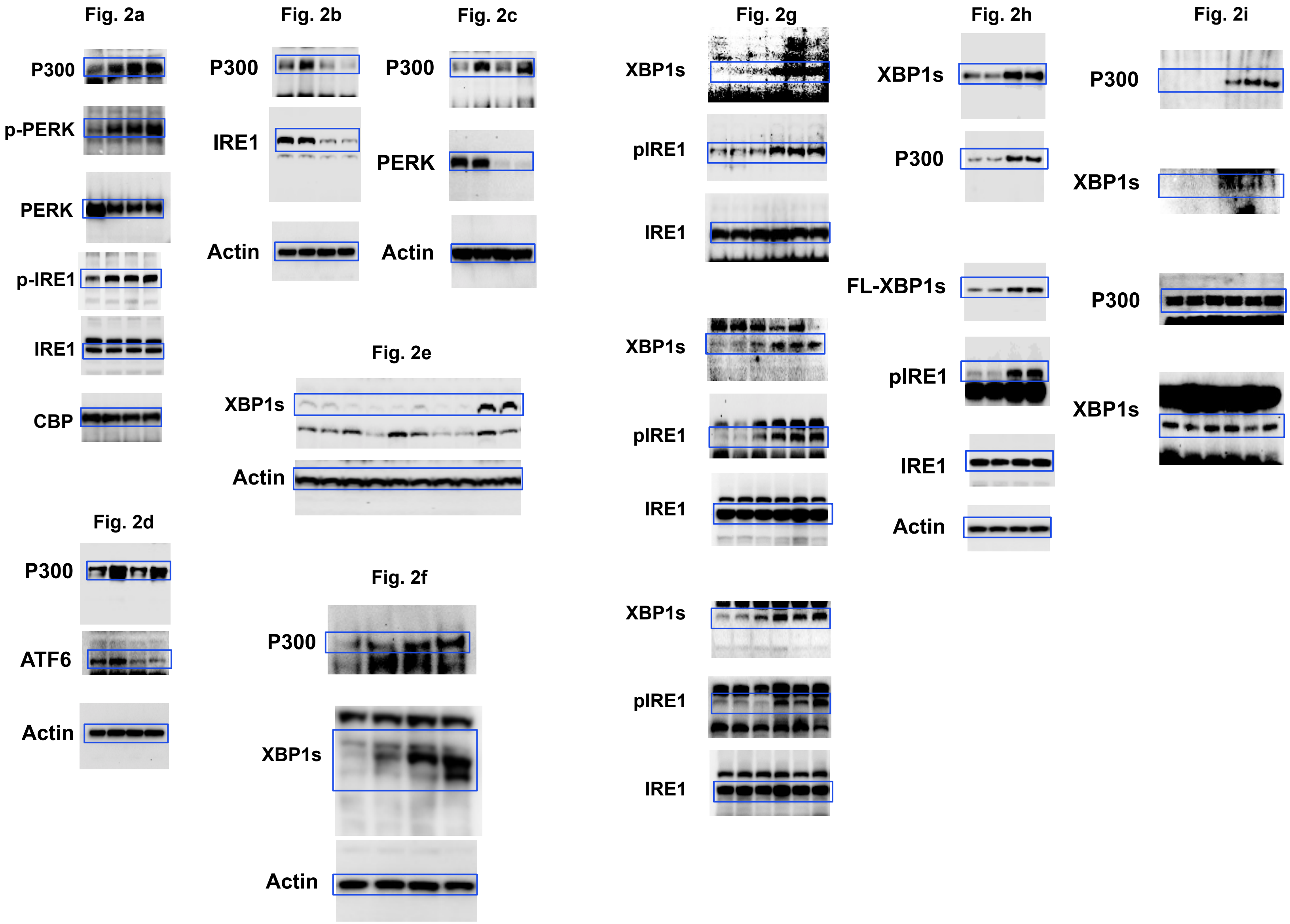

Supplementary Figure 17 Uncropped blots for Supplementary Fig. 2a-i. The bands used in the figure are marked in blue squares.

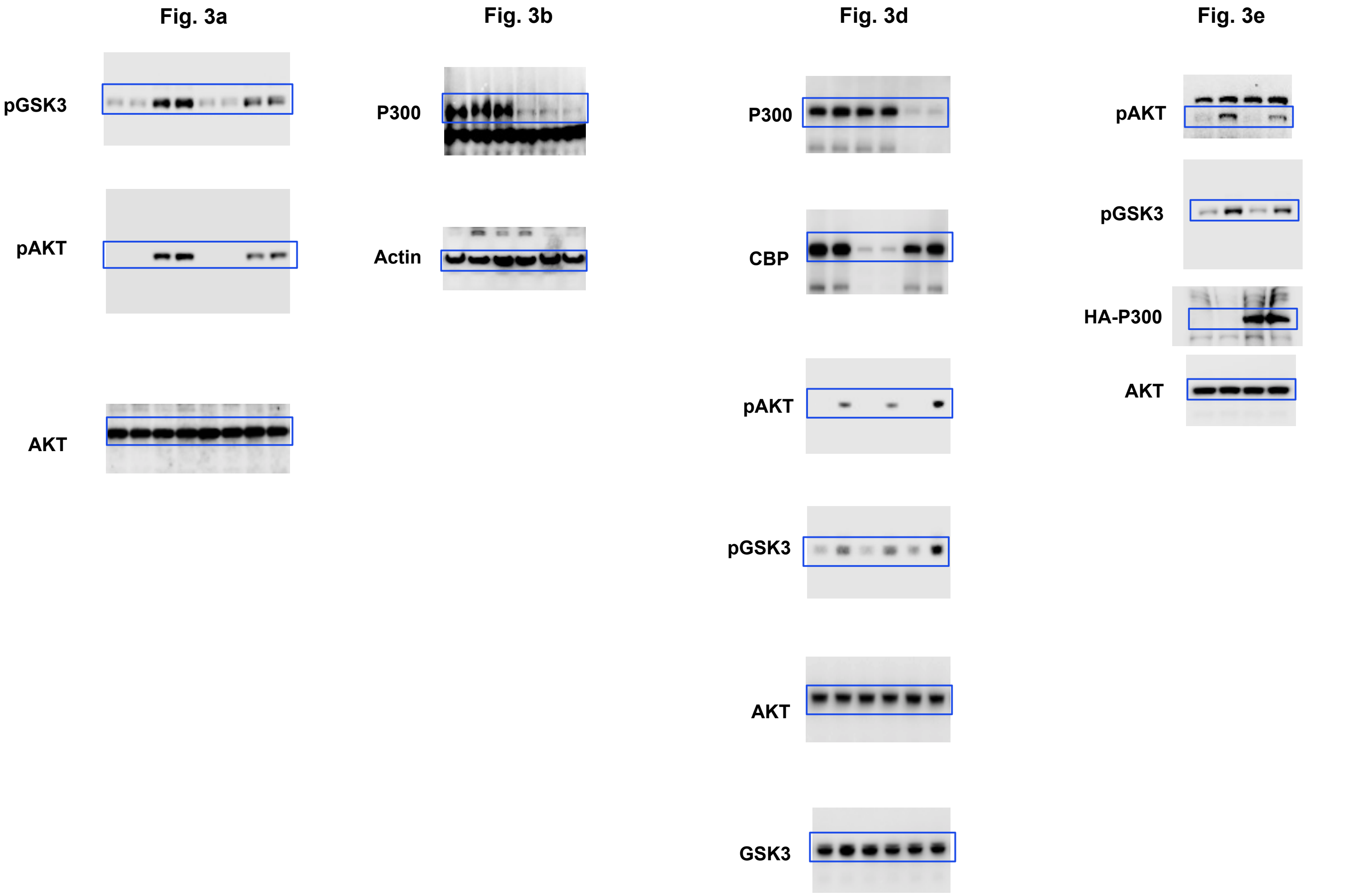

Supplementary Figure 18 Uncropped blots for Supplementary Fig. 3a, b, d, e. The bands used in the figure are marked in blue squares.

Fig. 4a

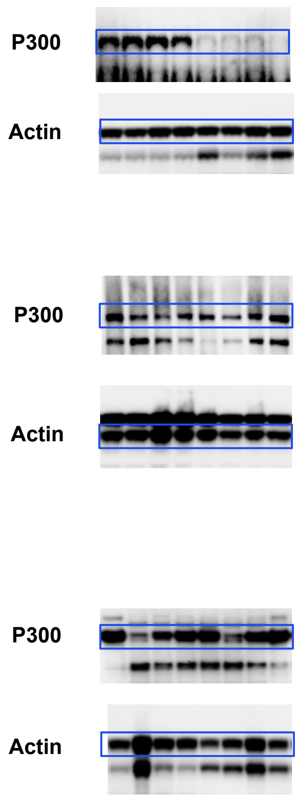

Supplementary Figure 19 Uncropped blots for Supplementary Fig. 4a. The bands used in the figure are marked in blue squares.

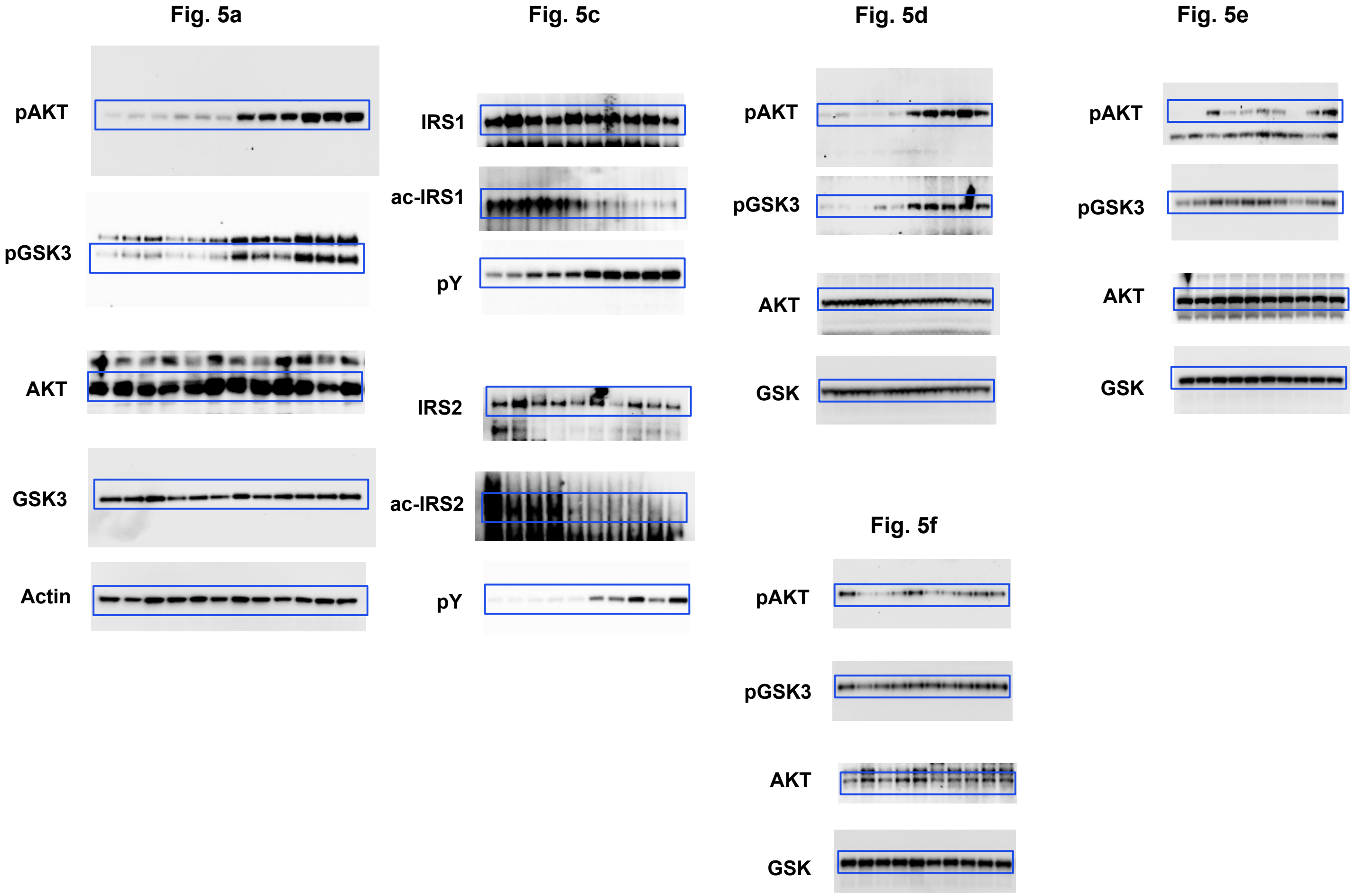

Supplementary Figure 20 Uncropped blots for Supplementary Fig. 5a, c-f. The bands used in the figure are marked in blue squares.

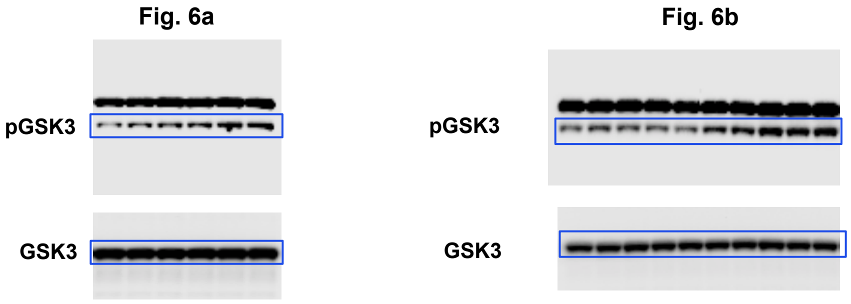

Supplementary Figure 21 Uncropped blots for Supplementary Fig. 6a, b. The bands used in the figure are marked in blue squares.

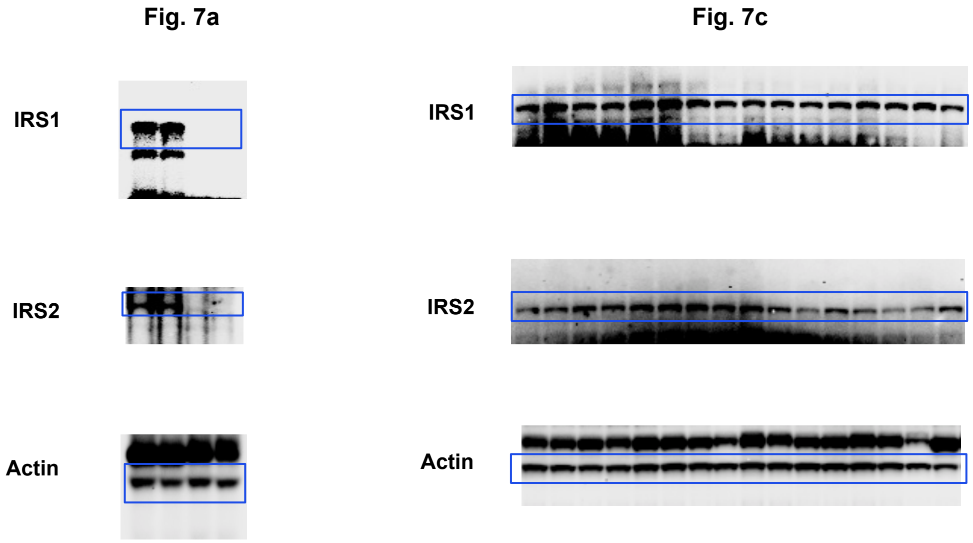

Supplementary Figure 22 Uncropped blots for Supplementary Fig. 7a, c. The bands used in the figure are marked in blue squares.
